# Integrating multiomics to elucidate the role of chromatin remodeling in glioma and the antitumor mechanisms and therapeutic potential of targeting LMNA

**DOI:** 10.3389/fimmu.2026.1759811

**Published:** 2026-05-08

**Authors:** Kun Wang, Feini Xu, Lingjie Zhu, Jiaying Wang, Haibo Li, Xianwen Cao, Xuhan Wang, Da Zong, Yuankun Liu, Junfei Shao

**Affiliations:** 1 Department of Neurosurgery, The Affiliated Wuxi People’s Hospital of Nanjing Medical University, Wuxi, China; 2 Wuxi Medical Center, The Affiliated Wuxi People’s Hospital of Nanjing Medical University, Wuxi, China; 3 Department of Dermatology, Affiliated Children’s Hospital of Jiangnan University (Wuxi Children’s Hospital), Wuxi, China; 4 Center of Clinical Research, The Affiliated Wuxi People’s Hospital of Nanjing Medical University, Wuxi, China; 5 Department of Neurosurgery, The Affiliated Yixing Hospital of Jiangsu University, Yixing, China

**Keywords:** chromatin remodeling, glioma, LMNA, machine learning, prognostic model

## Abstract

**Background:**

Glioma is a highly aggressive central nervous system malignancy characterized by poor clinical outcomes. While chromatin remodeling is fundamentally linked to tumorigenesis, its precise roles and underlying mechanisms in glioma remain elusive. Consequently, developing robust prognostic tools and identifying viable therapeutic targets are critical priorities.

**Methods:**

Leveraging RNA-seq profiles from the CGGA, GTEx, and TCGA cohorts, we identified chromatin remodeling-related genes (CRRGs) differentially expressed between glioma and normal brain tissues. We stratified patients into distinct molecular subtypes using consensus clustering and engineered a prognostic signature by systematically screening 117 machine-learning algorithm combinations. The expression patterns of core signature genes were cross-validated using single-cell RNA sequencing, spatial transcriptomics, and proteomic data. Finally, we interrogated the functional role of LMNA through *in vitro* and *in vivo* assays, complementing these studies with virtual screening to identify potential LMNA-targeting compounds.

**Results:**

Consensus clustering segregated gliomas into two CRRG-based subtypes, with Subgroup 2 patients experiencing significantly better prognoses. We pinpointed 28 glioma-specific DECRRGs and derived an optimal SuperPC-based model that robustly stratified patients into high- and low-risk cohorts. LMNA, a core model gene markedly upregulated in glioma and correlated with poor survival, was advanced for experimental validation. Functionally, LMNA depletion severely impaired tumor cell proliferation and invasion *in vitro*, and potently suppressed tumor growth *in vivo*. Furthermore, molecular docking analyses identified four natural compounds as candidate LMNA inhibitors.

**Conclusion:**

The CRRG-based prognostic signature developed herein demonstrates robust clinical utility for risk stratification. Crucially, our findings highlight LMNA as a potential prognostic biomarker and a promising therapeutic target in glioma.

## Graphical Abstract

**Figure:**
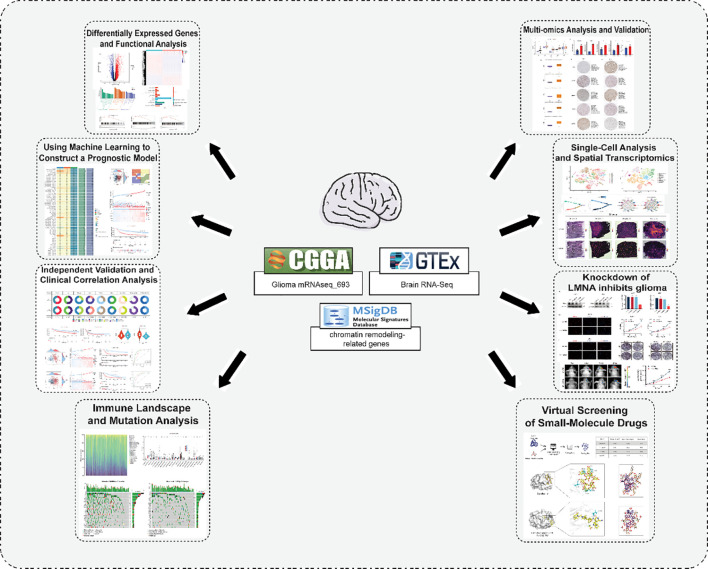

## 1 Introduction

Glioma, the most prevalent primary intracranial tumor originating from neuroglial progenitor cells, predominantly afflicts the adult population (1). Comprising approximately 27.5% of all central nervous system (CNS) tumors and up to 75% of malignant brain neoplasms, gliomas are characterized by relentless invasive growth, profound molecular heterogeneity, and formidable resistance to standard therapies (2). These features culminate in dismal clinical outcomes. Glioblastoma (GBM), the most aggressive subtype, carries a 5-year survival rate of less than 10% and is responsible for over 60% of adult CNS tumor-related mortality. While the 2021 WHO classification stratifies these tumors by molecular and histological features into grades 2, 3, and 4 (3), the current standard of care—maximal safe cytoreductive surgery followed by chemoradiotherapy (4) —yields marginal survival benefits, with a median survival of less than 15 months (5, 6). Even lower-grade gliomas (LGGs) frequently resist conventional interventions and inevitably progress (7). Although recent advances have illuminated the genetic and epigenetic drivers of gliomagenesis, spurring interest in targeted and immune-based therapies (8), and have established the tumor microenvironment (TME) as a critical facilitator of immune evasion (9), the overarching regulatory networks remain incompletely mapped. This gap underscores an urgent need to identify key molecular vulnerabilities to better understand disease biology and design more effective treatments.

Chromatin remodeling dictates the dynamic architectural shifts of the epigenome, orchestrating transitions between transcriptionally silent condensed states and accessible open conformations. This intricate process is mediated by both covalent epigenetic modifications (e.g., DNA methylation and histone tail alterations) and the noncovalent, ATP-dependent mobilization of nucleosomes by multisubunit complexes, such as SWI/SNF, INO80, and ISWI (10, 11). By modulating the accessibility of genomic loci to transcriptional machinery, chromatin remodelers govern gene expression landscapes essential for cellular homeostasis. Consequently, aberrations in these remodeling networks are hallmarks of oncogenesis, driving genomic reprogramming, tumor evolution, and therapy resistance (12, 13). For instance, the chromatin remodeling factor ARID2 suppresses hepatocellular carcinoma metastasis by recruiting DNMT1 to the *SNAIL* promoter, thereby silencing its transcription via targeted DNA methylation (14). Similarly, pharmacological modulation of chromatin architecture—such as HDAC6 inhibition by Nexturastat A—can reprogram the transcriptional landscape of triple-negative breast cancer (TNBC) cells to unleash anti-tumor immune responses (15).

Despite these insights, the precise regulatory mechanisms governed by chromatin remodeling in glioma remain poorly characterized. To bridge this knowledge gap, we integrated multi-omics data with a rigorous machine-learning framework to comprehensively profile chromatin remodeling-related genes (CRRGs) and construct a robust prognostic model for glioma patients. Furthermore, we experimentally validated the pivotal oncogenic role of the core signature gene, *LMNA*, in glioma initiation and progression through comprehensive *in vitro* and *in vivo* assays.

## 2 Materials and methods

### 2.1 Data collection and normalization

RNA-seq data and corresponding clinical annotations for glioma tumor tissues were acquired from the Chinese Glioma Genome Atlas (CGGA, https://www.cgga.org.cn/; accessed May 12, 2025) (16–19), comprising the CGGA_325 and CGGA_693 cohorts. Normal brain tissue RNA-seq profiles were obtained from the Genotype-Tissue Expression (GTEx) portal (https://www.gtexportal.org/home/; accessed May 12, 2025), specifically targeting glioma-prone regions such as the cortex, anterior cingulate cortex, caudate, and nucleus accumbens. Additional gene expression matrices and clinical data for low-grade glioma (LGG) and glioblastoma (GBM) cohorts with available survival records were downloaded from the UCSC Xena platform (https://xenabrowser.net/datapages/; accessed May 25, 2025) (20) and the GEO database (GSE43378, GPL570 platform; accessed May 23, 2025). After filtering out rows and columns with over 50% missing values, the expression data were log2(x+1) transformed, and all gene identifiers were standardized to official gene symbols.

Ultimately, 406 chromatin remodeling-related genes (CRRGs) were compiled from the Molecular Signatures Database (MSigDB v2025.1.Hs; accessed May 26, 2025). Briefly, gene sets explicitly containing the term “Chromatin Remodeling” in their titles or descriptions (restricted to *Homo sapiens*) were manually curated, pooled, and deduplicated. To ensure absolute reproducibility against future database updates, the definitive, non-redundant list of the 406 CRRGs utilized in all subsequent analyses is provided in **Supplementary Table 1** (21–23).

### 2.2 Screening of DEGs in glioma

Differentially expressed genes (DEGs) between glioma samples (CGGA_693 dataset) and normal brain tissues (GTEx) were identified using the “DESeq2” R package. The thresholds for statistical significance were strictly defined as |log_2_FC| > 1 and an adjusted p-value < 0.05. Visualizations, including heatmaps and volcano plots, were generated utilizing the “pheatmap” (v1.0.12) and “ggplot2” (v3.4.1) packages (24), respectively.

### 2.3 Consensus clustering based on CRRGs

To delineate distinct molecular subtypes based on CRRG expression, we performed unsupervised consensus clustering using the “ConsensusClusterPlus” R package (25). The analysis was applied to the expression profiles of the 406 CRRGs across the glioma cohorts. For each potential cluster count *k* (ranging from 2 to 10), the algorithm executed 1,000 iterations of subsampling, randomly selecting 80% of the samples per iteration. K-means clustering was performed utilizing Euclidean distance as the dissimilarity metric. A consensus matrix was constructed for each *k*, where each entry reflects the frequency of two samples clustering together across all iterations. Values approaching 0 or 1 denote stable co-clustering or consistent separation, respectively. The cumulative distribution function (CDF) of the consensus matrix was plotted for each *k*, and the area under the CDF curve was calculated to evaluate clustering stability. The optimal cluster count was determined by assessing the CDF curve inflection points and the relative area change, strictly adhering to established consensus clustering guidelines.

### 2.4 Enrichment analysis

Gene Ontology (GO) and Kyoto Encyclopedia of Genes and Genomes (KEGG) enrichment analyses were conducted via the “ClusterProfiler” R package to characterize the biological processes (BP), molecular functions (MF), cellular components (CC), and signaling pathways associated with the glioma subtypes. Results were visualized using Metascape. To detect subtle variations in pathway activity, we applied gene set variation analysis (GSVA), which translates gene-level expression into sample-specific enrichment scores, enabling the robust evaluation of functional pathway activity (26). Furthermore, gene set enrichment analysis (GSEA) was employed to compare predefined gene sets across different risk groups, providing a comprehensive functional landscape of transcriptional changes in glioma (21). The significance thresholds for GSEA were set at |NES| > 1, p < 0.05, and FDR < 0.25.

### 2.5 Construction of prognostic models via MIME-fused machine learning algorithms

To derive a robust prognostic signature, we employed MIME, a flexible machine-learning framework tailored for high-dimensional omics data (27). This framework integrates ten distinct algorithms (including Lasso, elastic net, random forest, CoxBoost, and SuperPC) to facilitate combined modeling and feature selection for survival analysis.

During the modeling phase, each algorithm was evaluated via 10-fold cross-validation within the CGGA_693 cohort. Model performance was benchmarked using Harrell’s C-index and time-dependent ROC curves for 1-, 3-, and 5-year overall survival. The optimal model, which utilized a SuperPC-driven ensemble strategy, demonstrated superior predictive accuracy and was designated as the final prognostic classifier. Hyperparameter optimization was seamlessly executed via MIME’s built-in grid-search capability. Model interpretability was subsequently enhanced by assessing feature importance, stratifying risk groups via Kaplan–Meier analysis, and conducting time-dependent ROC analysis. Briefly, all 117 algorithm combinations were trained on the CGGA_693 cohort via 10-fold cross-validation, and the final model was selected based on its average performance across two independent validation cohorts (TCGA_GBM and GSE108474).

### 2.6 Construction and validation of the nomogram

A prognostic nomogram integrating all independent predictive factors was constructed using the “rms” R package to estimate 1-, 3-, and 5-year survival probabilities. The predictive accuracy and calibration of the nomogram were comprehensively evaluated utilizing the concordance index (C-index) and calibration curves (accessed from https://CRAN.R-project.org/package=rms, June 23, 2025).

### 2.7 Tumor mutational burden analysis and immune-related functional analysis

Somatic mutation data from the TCGA were utilized to assess the tumor mutation burden (TMB) across glioma subgroups. Mutation profiles were processed using the “Maftools” R package to quantify and contrast variant frequencies among patient cohorts. Concurrently, transcriptomic data were evaluated using the ESTIMATE algorithm to calculate immune and stromal scores, reflecting the relative proportions of immune and stromal cell infiltration within the tumor microenvironment (28).

The relative abundances of 22 distinct immune cell subtypes were estimated for each tumor sample via CIBERSORT, a robust deconvolution algorithm that infers cell-type composition from bulk RNA expression profiles (29). Gene expression data, pre-normalized by the “limma” R package, were processed through the CIBERSORT platform (R script v1.03). Differential immune infiltration between high- and low-risk groups was statistically assessed based on these output abundance matrices. Immune functional profiles were additionally evaluated via single-sample gene set enrichment analysis (ssGSEA). Finally, to map associations between risk scores and differentially infiltrated immune cells, we performed Kendall correlation analysis, visualizing significant associations (p < 0.05) via a correlation lollipop plot.

### 2.8 Single-cell sequencing analysis

Single-cell RNA sequencing (scRNA-seq) data were processed and analyzed using the “Seurat” R package (30). Low-quality cells were excluded based on high proportions of mitochondrial or ribosomal transcripts. The raw count data were normalized utilizing the “NormalizeData” function, and the top 2000 highly variable genes were identified via “FindVariableFeatures”. Principal component analysis (PCA) was subsequently performed on these informative genes using the “RunPCA” function. Batch effects across different samples were corrected utilizing the “Harmony” algorithm, followed by dimensionality reduction and cluster visualization via uniform manifold approximation and projection (UMAP) (31). Pseudotime trajectory analysis was executed with the “monocle” package, employing the “DDRTree” method for nonlinear dimensional reduction to map cellular state transitions (32).

### 2.9 Cell culture and transfection

The human glioma cell lines U251 and U87 were procured from the Chinese Academy of Sciences (Shanghai, China) and authenticated via STR profiling. Cells were cultured in high-glucose Dulbecco’s Modified Eagle Medium (DMEM, Gibco) supplemented with 10% fetal bovine serum (FBS) and 1% penicillin/streptomycin in a humidified incubator.

To elucidate the functional role of LMNA, we established stable LMNA-knockdown cell lines using lentiviral-mediated short hairpin RNA (shRNA) delivery. The *LMNA* gene encodes two major A-type lamin isoforms, lamin A and lamin C, generated through alternative splicing. Our shRNA constructs were specifically designed to target regions shared by both isoforms, ensuring the simultaneous silencing of lamin A and lamin C. Three independent shRNAs targeting human *LMNA* (sh-LMNA1, sh-LMNA2, sh-LMNA3) and a non-targeting negative control (sh-NC) were cloned into the pLKO.1-puro lentiviral vector. U251 and U87 cells were seeded into 6-well plates at 5 × 10^4^ cells/well. After 24 hours, cells were transduced with lentiviral particles in the presence of 8 μg/mL polybrene for 24 hours, followed by replacement with complete medium. At 48 hours post-transduction, stable clones were selected using 2 μg/mL puromycin for 7–10 days. Knockdown efficiency was rigorously verified via quantitative real-time PCR and Western blotting (detailed in Sections 1.10 and 1.11). The construct yielding the most potent knockdown (sh-LMNA3) was exclusively utilized for all subsequent functional assays (33).

### 2.10 RNA isolation, reverse transcription and qRT–PCR

This study was approved by the Ethics Committee of Wuxi People’s Hospital (Approval No. 2024374). Total RNA was extracted from primary glioma cells using TRIzol reagent (Invitrogen) according to the manufacturer’s instructions. Complementary DNA (cDNA) was synthesized utilizing the PrimeScript RT Reagent Kit (TaKaRa). Quantitative real-time reverse transcription PCR (qRT-PCR) was performed on a LightCycler 480 II system (Roche) using real SYBR mixture (CWBIO, Beijing, China) to quantify mRNA expression levels. All experiments were performed with three independent biological replicates (n = 3). The specific primer sequences used were:

forward 5′-CCCCTCATCCCTAAACAGCA-3′ and reverse 5′-CGAAGGACAGAGACTGCTCG-3′;

The SPI1-specific primers used were as follows:

forward 5′-GCAGGGGATCTGACCGACT-3′ and reverse 5′-AAGCTCTCGAACTCGCTGTG-3′;

The LMNB1-specific primers used were as follows:

forward 5′-TCGAGAATATGAAGCAGCACTGA-3′ and reverse 5′-AAGGCTCTGACAACGATTCTCC-3′;

The LMNB2-specific primers used were as follows:

forward 5′-ACTACATCGACCGCGTCC-3′ and reverse 5′-CTTCTTGGCGCTCTTGTTGAC-3′;

The CBX8-specific primers used were as follows:

forward 5′-AAGCGGCGCATACGGAAAG-3′ and reverse 5′-GCTGAGTCACTTCGAAACTCG-3′.

### 2.11 Western blotting

Cells were lysed in RIPA buffer (Sigma–Aldrich, 20–188) under thermally denaturing conditions. Following trypsin digestion (Servicebio, G4004) to facilitate protein precipitation, pellets were resuspended in RIPA lysis buffer. Lysates were centrifuged, and the supernatant protein concentrations were determined using a BCA assay kit (Thermo Fisher, A55865). Equal amounts of protein (10 μg/lane) were resolved via SDS-PAGE, transferred onto PVDF membranes, and blocked with 5% nonfat milk (BD Biosciences, 232100) in TBST for 1 hour. Membranes were incubated overnight at 4 °C with primary antibodies—including LMNA (Proteintech, 10298-1-AP, diluted 1:500 in Antibody Dilution Buffer [Beyotime, P0023A])—followed by incubation with species-specific HRP-conjugated secondary antibodies (Promega, W4011 for rabbit; W4021 for mouse) diluted in TBST. GAPDH and Histone H3 were utilized as loading controls where appropriate. Protein bands were visualized using enhanced chemiluminescence (ECL) and quantified using ImageJ software. All Western blot experiments were performed with three independent biological replicates (n = 3).

### 2.12 Functional assay

#### 2.12.1 Cell proliferation assays

Proliferation was quantified using the CCK-8 assay. Cells were seeded into 96-well plates at 2,000 cells/well (n=3/group). At 24, 48, and 72 hours, the medium was replaced with a CCK-8 working solution (Solarbio, CA1210) and incubated for 1 hour at 37 °C in 5% CO_2_. Absorbance was recorded at 450 nm using a microplate reader. For colony formation assays, cells were seeded into 6-well plates (1,000 cells/well, n=3/group) and cultured for 144 hours. Colonies were subsequently fixed, stained with Giemsa (Beyotime, C0133), and manually counted. Proliferation was further corroborated using an EdU incorporation assay (KeyGen Biotech, China), where EdU-positive cells were visualized and quantified under a fluorescence microscope.

#### 2.12.2 Migration and invasion assays

For Transwell assays, cells were starved in serum-free medium for 2 hours. Subsequently, 2 × 10^4^ cells suspended in serum-free medium were seeded into the upper chamber of 24-well Transwell inserts (Corning) (n = 3 per group). For invasion assays, the upper chambers were pre-coated with Matrigel (BD Biosciences). The lower chambers contained medium supplemented with 10% FBS acting as a chemoattractant. Following a 72-hour incubation, non-migrating cells on the upper surface were removed with a cotton swab. Cells that successfully migrated or invaded the lower surface were fixed, stained with Giemsa, and counted microscopically. Wound healing assays were similarly employed to assess 2D migration. Cells were grown to confluence in 6-well plates (1 × 10^5^ cells/well, n = 3 per group), and a linear scratch was created using a 200 µL pipette tip. After washing with PBS to remove debris, cells were maintained in serum-free medium. Wound closure was documented at 0, 24, and 48 hours using an inverted microscope, and the open wound area was quantified via ImageJ software.

### 2.13 Glioma intracranial mouse model

All animal protocols complied with the National Institutes of Health Guide for the Care and Use of Laboratory Animals and were strictly approved by the Ethics Committee of Nanjing Medical University (Approval No. 2022108). Technicians conducting the *in vivo* measurements were blinded to the treatment groups. Four-week-old female BALB/c nude mice were sourced from the Shanghai Animal Center (Chinese Academy of Sciences) and maintained under specific pathogen-free (SPF) conditions. For the orthotopic xenograft model, U87 cells stably expressing firefly luciferase (transduced with sh-LMNA or sh-NC constructs) were suspended in a DMEM/Matrigel mixture (BD Biosciences). The cell suspension (5 × 10^5^ cells in 5 µL) was stereotactically injected into the right striatum of each mouse (n = 5 per group). Tumor progression was monitored longitudinally via *in vivo* bioluminescence imaging using an IVIS Spectrum system, with signals quantified via Living Image software. At the experimental endpoint, all animals were humanely euthanized by anesthetic overdose in strict accordance with ethical guidelines.

### 2.14 Virtual screening and molecular docking

The 3D structure of the LMNA domain was acquired from the RCSB Protein Data Bank (https://www.rcsb.org; accessed May 28, 2025) (34). Virtual screening was conducted against the MCE Natural Product Library—a collection of natural small-molecule compounds comprising primary and secondary metabolites with potential bioactivity. Utilizing a locally deployed KarmaDock pipeline, molecular docking was systematically performed for each compound in the library against the LMNA target structure to generate corresponding docking scores. High-scoring hit molecules were subsequently analyzed, and their predicted binding modes with LMNA were rendered in 3D using PyMOL software.

### 2.15 Statistical analysis

Statistical analyses were performed using GraphPad Prism (version 8.4.3) and R software (version 4.2.1). For two-group comparisons, the selection between parametric (Student’s t-test) and nonparametric (Wilcoxon rank-sum test) methods was dictated by data distribution. Normality was verified via the Shapiro–Wilk test (p > 0.05 indicating a normal distribution), and variance homogeneity was assessed using Levene’s test. If both assumptions were met, the Student’s t-test was applied; otherwise, the Wilcoxon test was utilized. For multi-group comparisons, a one-way ANOVA (parametric) or Kruskal–Wallis test (nonparametric) was employed, followed by appropriate *post-hoc* tests incorporating multiple comparison corrections (e.g., Tukey’s or Dunn’s test). A p-value < 0.05 was considered statistically significant. These rigorous statistical frameworks ensure that the analytical methods align with the underlying data distributions, thereby maximizing the robustness and reproducibility of our findings.

## 3 Results

### 3.1 Analysis of differentially expressed genes and identification of CRRG-based subtypes in glioma

**Figure 1:**
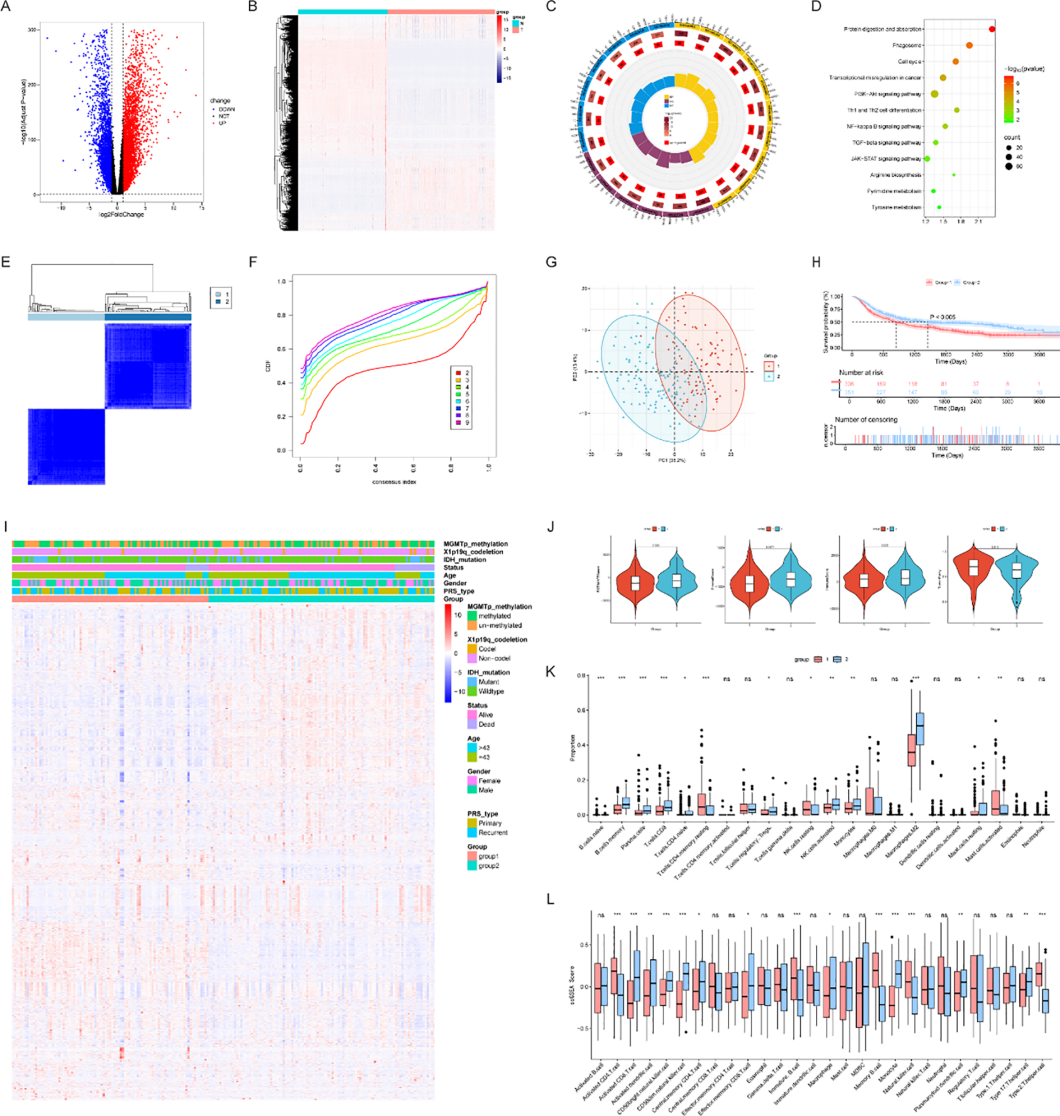
Analysis of differentially expressed genes (DEGs) and identification of CRRG-based subtypes in glioma. **(A)** Volcano plot of DEGs between glioma and normal brain tissues; **(B)** Heatmap showing the expression patterns of DEGs that distinguish gliomas from normal tissues; **(C)** Circular plot showing the Gene Ontology (GO) enrichment analysis of DEGs in glioma; **(D)** Bubble chart showing the Kyoto Encyclopedia of Genes and Genomes (KEGG) pathway enrichment analysis of DEGs in glioma; **(E)** Consensus matrix of unsupervised clustering (k=2) based on CRRGs; **(F)** Cumulative distribution function (CDF) curve for different numbers of clusters; **(G)** Principal component analysis (PCA) plot showing the separation of two CRRG-based subtypes (Group 1, Group 2); **(H)** Kaplan–Meier survival curve for overall survival (OS) between Group 1 and Group 2; **(I)** Heatmap of DEG expression and clinical feature subgroups in glioma; **(J)** Violin plots of stromal scores, immune scores, ESTIMATE scores, and tumor purity between subtypes; **(K, L)** Boxplots detailing the immune infiltration differences between subtypes analyzed by CIBERSORT **(K)** and ssGSEA **(L)** (ns, not significant; *p < 0.05, **p < 0.01, ***p < 0.001).

To characterize transcriptomic alterations and identify chromatin remodeling-related subtypes in glioma, we first compared the RNA-seq profiles of tumor samples with those of normal brain tissues. Integrating data from the CGGA and GTEx cohorts, we identified 11,117 differentially expressed genes (DEGs), comprising 6,629 upregulated and 4,488 downregulated genes (|log_2_FC| > 1, adjusted p < 0.05). The distribution of these DEGs is illustrated in the volcano plot (**Figure 1A**). Furthermore, the corresponding heatmap (**Figure 1B**) demonstrates a stark transcriptional separation between glioma and normal brain tissues, corroborating the presence of robust disease-specific expression signatures.

To elucidate the biological significance of these DEGs, we performed Gene Ontology (GO) and Kyoto Encyclopedia of Genes and Genomes (KEGG) enrichment analyses. GO analysis revealed a predominant enrichment in biological processes related to immune regulation [e.g., T cell activation (GO:0050863), lymphocyte proliferation (GO:0046651), and macrophage activation (GO:0042116)], cellular secretion [e.g., regulated exocytosis (GO:0099501)], and extracellular matrix organization (GO:0005201). Molecular functions such as cytokine and immune receptor activity were also highly enriched (**Figure 1C**). Similarly, KEGG pathway analysis implicated the DEGs in protein and pyrimidine metabolism, transcriptional dysregulation, and critical oncogenic signaling cascades, including the NF-κB, PI3K, and JAK-STAT pathways (**Figure 1D**).

We subsequently performed unsupervised consensus clustering on 406 chromatin remodeling-related genes (CRRGs) curated from the Molecular Signatures Database (MSigDB). Utilizing the “ConsensusClusterPlus” algorithm across 1,000 iterations, we generated consensus matrices and cumulative distribution function (CDF) curves for cluster numbers *k* = 2 to 10. Evaluation of these metrics identified *k* = 2 as the optimal and most stable configuration, characterized by a clear block-diagonal consensus matrix and a CDF curve that plateaued with minimal subsequent area expansion (**Figures 1E, F**). At this threshold, samples sharply segregated into two distinct modules. By contrast, higher-*k* solutions (*k* = 3 and 4) yielded ambiguous boundaries and substantial cluster overlap, indicating insufficient separation. Critically, survival analysis across these higher-*k* partitions revealed no significant prognostic differences (**Supplementary Figures 1A–F**), suggesting they offered no additional clinical utility beyond the bipartite model. Consequently, the *k* = 2 solution was adopted. The two resulting subtypes (Subgroup 1 and Subgroup 2) were visually well-separated in the dimensionality reduction projection (**Figure 1G**) and exhibited a highly significant difference in overall survival (**Figure 1H**), with patients in Subgroup 2 experiencing markedly better outcomes. This robust stratification confirms that CRRG expression patterns are fundamentally intertwined with patient survival. The subtype heatmap (**Figure 1I**) aligns these DEG expression profiles with key clinical characteristics, including IDH mutation status, 1p/19q codeletion, and WHO grade.

ESTIMATE analysis demonstrated that patients in Subgroup 2 possessed notably higher stromal, immune, and overall tumor microenvironment scores (**Figure 1J**). To further dissect the immune contexture, we applied CIBERSORT and single-sample gene set enrichment analysis (ssGSEA). CIBERSORT profiling revealed significantly elevated abundances of B cells, T cells, and M2-type macrophages in Subgroup 2 compared to Subgroup 1 (**Figure 1K**). Consistent with these findings, ssGSEA confirmed broader immune cell infiltration in Subgroup 2. However, subtype-specific nuances were observed; for instance, NK cells were more enriched in Subgroup 1, whereas NKT cell levels showed no significant disparity between the two cohorts (**Figure 1L**).

### 3.2 Differentially expressed genes and functional annotations among CRRG-based glioma subtypes

**Figure 2:**
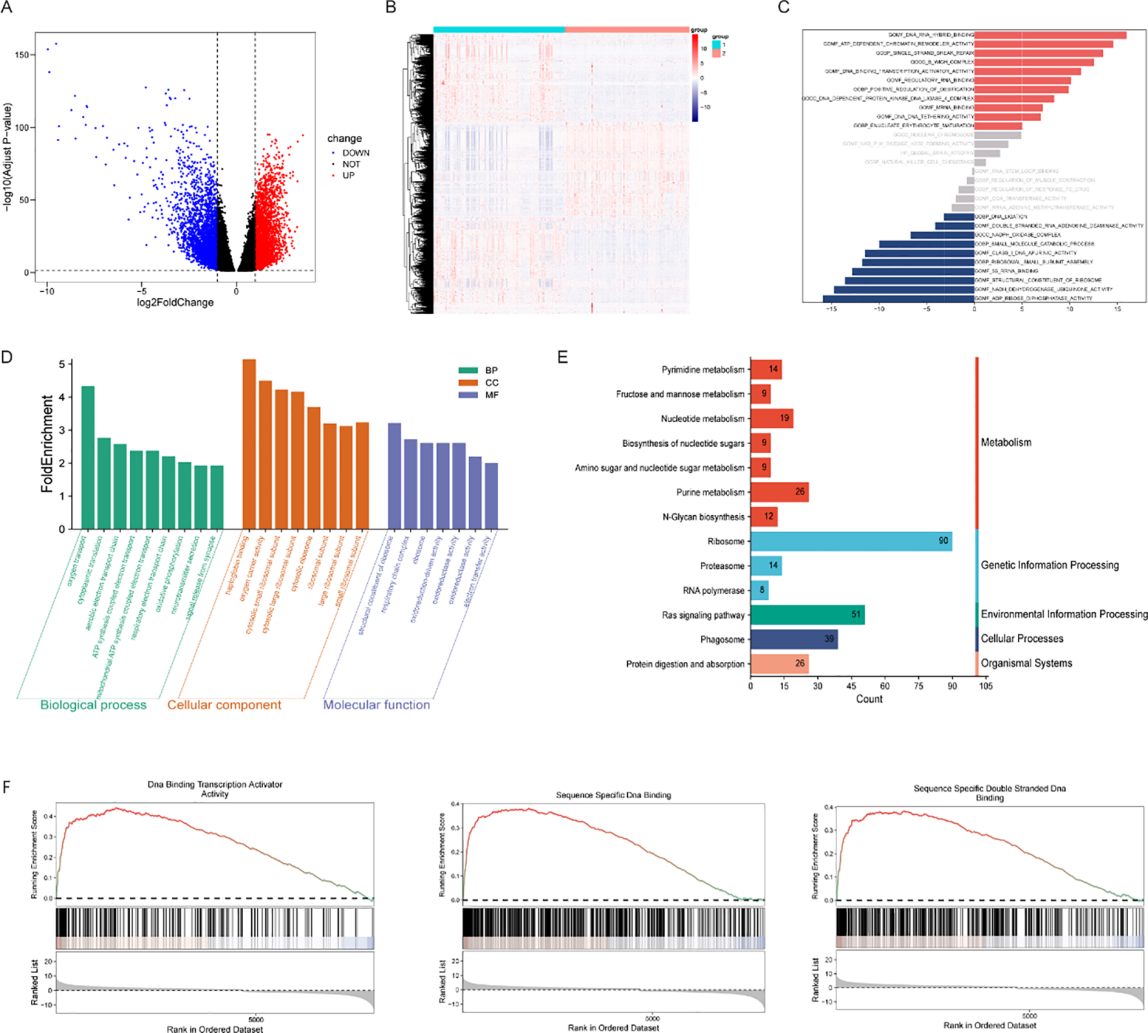
Differentially expressed genes and functional annotation in glioma patients with CRRG-based subtypes. **(A)** Volcano plot of DEGs between the Group 1 and Group 2 subtypes; **(B)** Heatmap of DEG expression patterns distinguishing the two subtypes; **(C)** Bar chart of Gene set variation analysis (GSVA) showing differences in functional activity between the two subtypes; **(D)** Bar chart illustrating the Gene Ontology (GO) enrichment analysis of DEGs; **(E)** Bar chart illustrating the Kyoto Encyclopedia of Genes and Genomes (KEGG) pathway enrichment analysis of DEGs; **(F)** GSEA enrichment plots showing the enrichment of functions related to DNA binding and chromatin remodeling among the DEGs.

To further delineate the molecular distinctions between the Subgroup 1 and Subgroup 2 subtypes, we performed differential expression analysis on their respective transcriptomic profiles. This analysis identified a total of 5,687 differentially expressed genes (DEGs) across the glioma cohorts (|log_2_FC| > 1, adjusted p < 0.05). Among these, 2,930 genes were upregulated (exhibiting higher expression in Subgroup 1), and 2,745 genes were downregulated (exhibiting higher expression in Subgroup 2) (**Figures 2A, B**).

Subsequent Gene Ontology (GO) and Kyoto Encyclopedia of Genes and Genomes (KEGG) enrichment analyses were conducted to elucidate the functional landscapes of these DEGs. GO annotation indicated a primary enrichment in processes associated with ribosomal architecture (including both large and small subunits) and redox homeostasis (**Figure 2D**). Concurrently, KEGG profiling highlighted the involvement of these DEGs in nucleotide metabolism (specifically purines and pyrimidines), ribosome biogenesis, and the Ras signaling cascade (**Figure 2E**).

To capture broader functional pathway shifts, we applied gene set variation analysis (GSVA) and gene set enrichment analysis (GSEA). GSVA demonstrated that pathways driving DNA–RNA binding, DNA-binding transcription activator functions, and chromatin remodeling were significantly upregulated in Subgroup 1. Conversely, signatures related to RNA composition and NADPH-linked redox activities were notably elevated in Subgroup 2 (**Figure 2C**). Furthermore, GSEA corroborated these trends, showing that core mechanisms underlying transcriptional regulation and chromatin remodeling—specifically DNA-binding transcription activator activity, as well as sequence-specific single- and double-stranded DNA binding—were highly enriched (**Figure 2F**).

### 3.3 Construction of 117 machine learning-based prognostic models for differentially expressed chromatin remodeling-related genes in glioma

**Figure 3:**
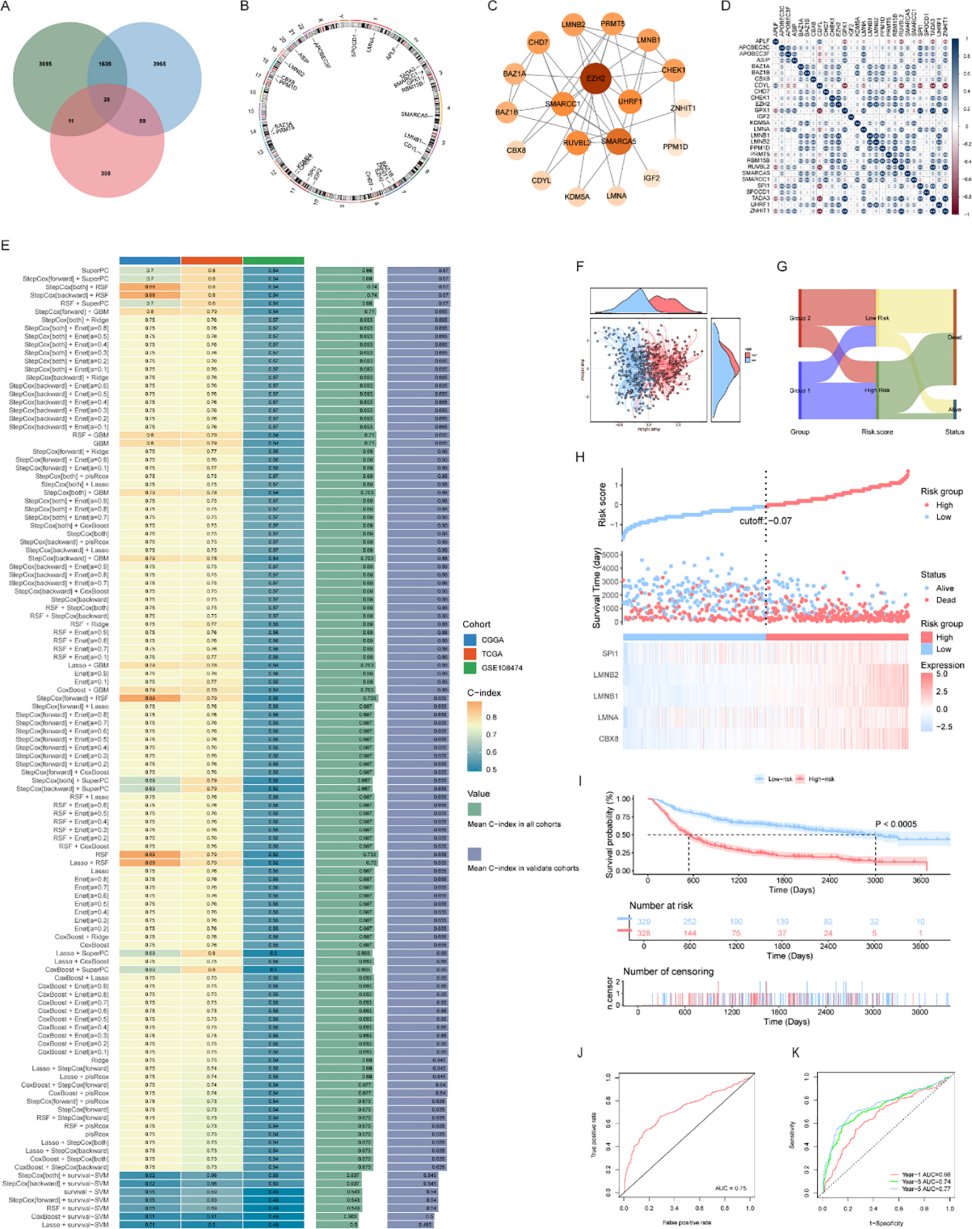
Construction and validation of machine learning prognostic models based on glioma DECRRGs. **(A)** Venn diagram showing the intersection of three gene sets: differentially expressed genes (DEGs) between glioma and normal tissues, DEGs between Group 1 and Group 2 subtypes, and chromatin remodeling-related genes (CRRGs). The overlap yielded 28 glioma-specific DECRRGs, which were used for subsequent analyses; **(B)** Circos plot showing the chromosomal distribution of the 28 glioma DECRRGs; **(C)** Protein‒protein interaction (PPI) network of proteins encoded by the 28 DECRRGs; **(D)** Correlation heatmap of the 28 DECRRGs (*p < 0.05, **p < 0.01); **(E)** Performance comparison of 117 combined algorithms generated by 10 machine learning approaches (using the C-index as the evaluation metric) to screen the optimal prognostic model; **(F)** Scatter plot of risk scores combined with survival status, showing the distribution of patients in the high- and low-risk groups; **(G)** Sankey diagram presenting the associations among subtype grouping, risk grouping, and patient survival status; **(H)** Scatter plot of risk scores and survival time, along with a heatmap of model-related gene expression in the high- and low-risk groups; **(I)** Kaplan–Meier survival curve of overall survival (OS) for patients in the high- and low-risk groups; **(J)** ROC curve for the overall prognostic prediction of the model; **(K)** Time-dependent ROC curves for 1-year, 3-year, and 5-year survival prediction.

To identify DECRRGs uniquely implicated in glioma, we intersected three distinct gene sets: DEGs between tumor and normal tissues, DEGs between the Subgroup 1 and Subgroup 2 subtypes, and the curated list of CRRGs. This intersection yielded a core panel of 28 glioma-specific DECRRGs (**Figure 3A**), whose chromosomal localizations were mapped via a Circos plot (**Figure 3B**). Subsequent protein-protein interaction (PPI) network analysis highlighted intricate functional and physical interactions among these targets (**Figure 3C**), while a correlation heatmap underscored highly significant co-expression patterns across the 28 genes (**Figure 3D**).

To construct a robust prognostic signature, we systematically evaluated 117 model configurations derived from 10 machine learning algorithms using 10-fold cross-validation. These models were trained on the CGGA_693 cohort and rigorously assessed across two independent validation cohorts (TCGA_GBM and GSE108474). By calculating the average C-index for each algorithm combination across both validation datasets (**Figure 3E**), we identified the SuperPC model as the optimal classifier. This model exhibited consistently superior predictive accuracy and stability across all three independent datasets and was consequently selected as the final prognostic signature (**Supplementary Figure 2**). This strict, external validation-driven screening pipeline effectively minimized potential biases stemming from overfitting or chance selection.

Based on the SuperPC signature, risk scores successfully stratified glioma patients into high- and low-risk cohorts (**Figure 3F**). A Sankey diagram visually confirmed that both CRRG subtype and risk stratification were tightly linked to clinical outcomes, with mortality overwhelmingly concentrated in the high-risk group (**Figure 3G**). Furthermore, survival scatter plots and expression heatmaps demonstrated a clear trajectory: as risk scores escalated, patient mortality rates and the expression levels of the core signature genes concomitantly increased (**Figure 3H**). Kaplan-Meier survival analysis definitively showed that patients in the high-risk cohort experienced markedly inferior prognoses compared to their low-risk counterparts (**Figure 3I**). Finally, time-dependent receiver operating characteristic (ROC) analyses were employed to benchmark prognostic accuracy. The model achieved an area under the curve (AUC) of 0.75 for overall survival, with AUC values of 0.66, 0.74, and 0.77 for 1-, 3-, and 5-year survival, respectively (**Figures 3J, K**), confirming its robust and reliable predictive performance.

### 3.4 Independent validation of the prognostic model

**Figure 4:**
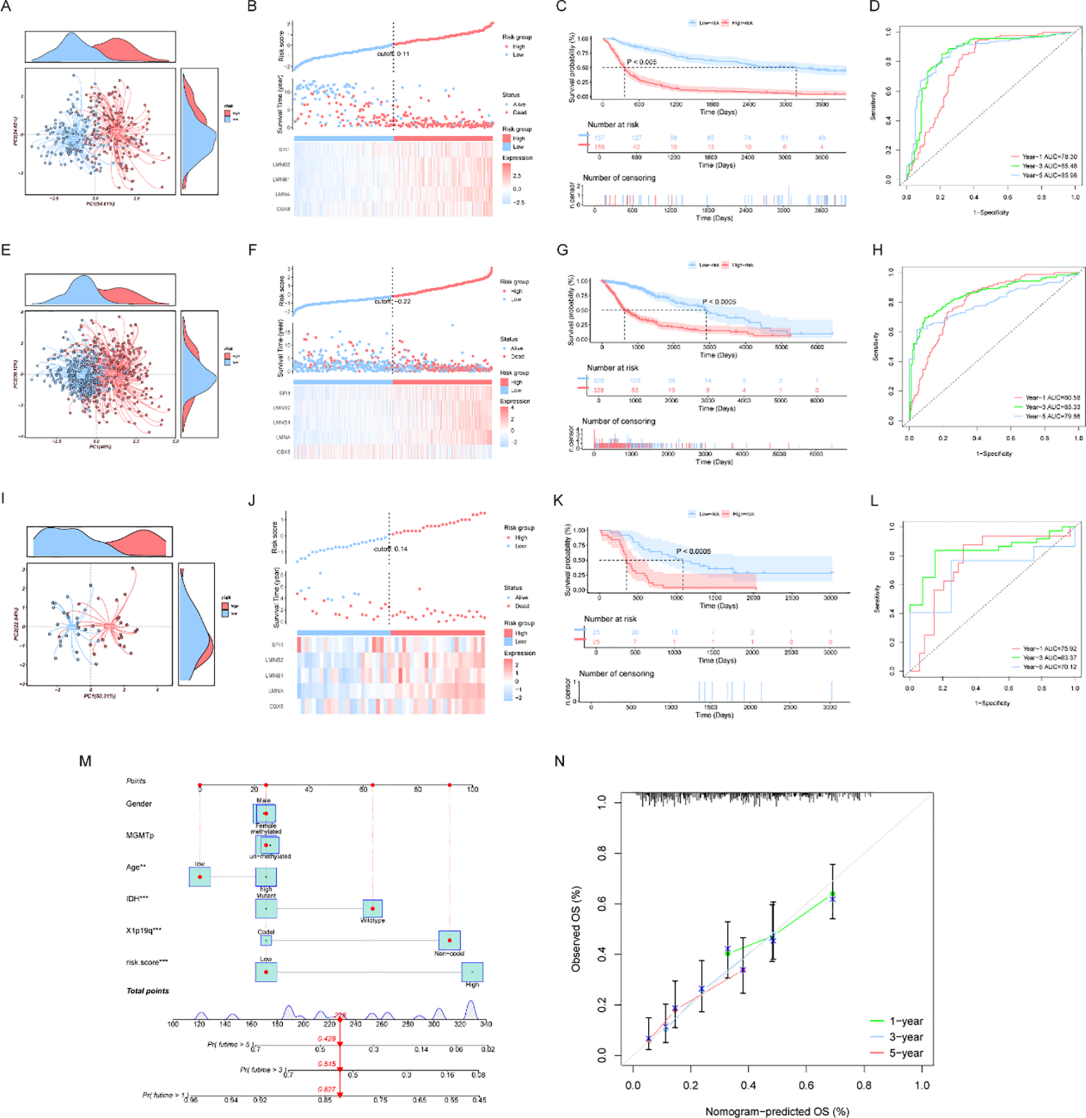
External validation of the DECRRG-based prognostic model. **(A, E, I)** Principal component analysis (PCA) plots of the CGGA_325, TCGA, and GSE43378 datasets; **(B, F, J)** Scatter plots of risk scores and survival status, paired with heatmaps of core gene expression in the CGGA_325, TCGA, and GSE43378 datasets; **(C, G, K)** Kaplan–Meier survival curves of the high- and low-risk groups in the CGGA_325, TCGA, and GSE43378 datasets, illustrating prognostic differences between the two groups; **(D, H, L)** ROC curves and time-dependent ROC curves of the CGGA_325, TCGA, and GSE43378 datasets, evaluating the predictive performance of the model; **(M)** Prognostic nomogram for the personalized prediction of 1-, 3-, and 5-year survival probabilities (ns, not significant; *p < 0.05, **p < 0.01, ***p < 0.001); **(N)** Calibration plots of the nomogram, reflecting the consistency between observed and predicted survival.

To rigorously verify the broad applicability and accuracy of the established prognostic model, we utilized three independent datasets (CGGA_325, TCGA, and GSE43378) to conduct principal component analysis (PCA), risk score-based correlation analysis, survival analysis, ROC analysis, and time-dependent ROC analysis. Across all three validation cohorts, PCA results indicated that the prognostic signature effectively stratified glioma patients into two transcriptionally distinct clusters: the high-risk and low-risk groups (**Figures 4A, E, I**).

Scatter plots clearly illustrated the trajectory of patient survival status in relation to risk scores: as risk scores escalated, patient mortality rates progressively increased. Concurrently, the expression levels of the core signature genes exhibited an upward trend alongside the rising risk scores (**Figures 4B, F, J**). Furthermore, Kaplan-Meier survival curves confirmed pronounced prognostic disparities between the two risk strata, with patients in the low-risk group experiencing significantly better overall survival compared to those in the high-risk group (**Figures 4C, G, K**). Evaluation via standard and time-dependent ROC analyses demonstrated that the model maintained robust predictive performance across these diverse independent datasets (**Figures 4D, H, L**; **Supplementary Figures 3A–C**).

To facilitate clinical translation and enable the personalized prediction of 1-, 3-, and 5-year survival probabilities for individual glioma patients, we constructed a prognostic nomogram integrating the risk score with other relevant clinical features (**Figure 4M**). Subsequently, calibration plots were generated to assess the nomogram’s predictive reliability. The resulting calibration curves exhibited excellent consistency between the nomogram-predicted probabilities and the actual observed overall survival rates, supporting its potential clinical utility (**Figure 4N**).

### 3.5 Clinical correlation analysis and independent validation of the prognostic model

**Figure 5:**
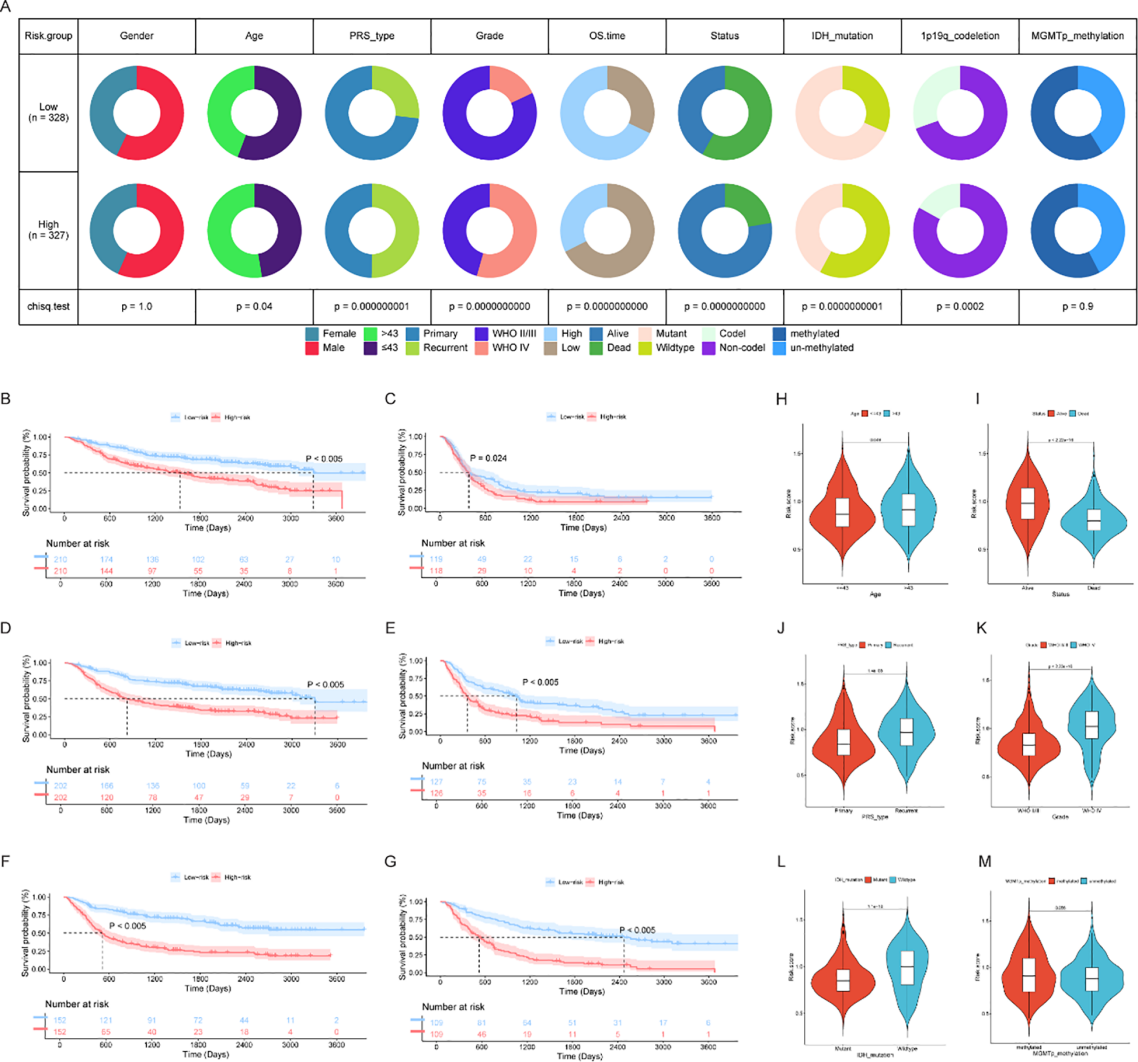
Clinical correlation analysis and independent validation of the prognostic model. **(A)** Donut charts illustrating the distribution differences and statistical significance of clinical features between the high-risk and low-risk groups, including sex, age, disease recurrence type, WHO grade, overall survival (OS) time, survival status, IDH mutation, 1p/19q codeletion, and MGMT methylation; **(B, C)** Survival curves of high-risk and low-risk patients in different WHO grade subgroups; **(D, E)** Survival curves of high-risk and low-risk patients in different disease recurrence subgroups; **(F, G)** Survival curves of high-risk and low-risk patients in different MGMT methylation status subgroups; **(H–M)** Violin plots showing the distribution of risk scores in subgroups with different clinical features (age, disease recurrence type, WHO grade, survival status, IDH mutation status, and 1p/19q codeletion status).

To further explore the intricate relationships between risk scores and diverse clinical features, and to validate the independent prognostic value of the developed CRRG-based model, we conducted rigorous subgroup and clinical correlation analyses. A circos plot was generated to visualize the differential distribution of clinical characteristics—including sex, age, disease recurrence type, WHO grade, overall survival (OS) time, survival status, IDH mutation, 1p/19q codeletion, and MGMT methylation—between the high- and low-risk groups (**Figure 5A**). Statistical evaluations revealed no significant differences in sex (p = 1.0) or MGMT methylation status (p = 0.9) between the two risk strata. In contrast, significant disparities were observed regarding age (p = 0.04), disease recurrence type (p = 1×10^−7^), WHO grade (p = 1×10^−7^), OS time (p = 1×10^−7^), survival status (p = 1×10^−7^), IDH mutation status (p = 1×10^−7^), and 1p/19q codeletion status (p = 0.0002). These findings imply a strong association between the risk classification and these key clinicopathological features.

To verify the prognostic utility of the model across specific clinical sub-cohorts, we performed stratified subgroup survival analyses. The results demonstrated that within diverse subgroups defined by age (**Supplementary Figures 4B, C**, p < 0.05), WHO grade (**Figures 5B, C**), recurrence type (**Figures 5D, E**), MGMT methylation status (**Figures 5F, G**), and IDH mutation status (**Supplementary Figures 4D, E**), patients in the high-risk group consistently exhibited markedly shorter OS compared to those in the low-risk group. These results indicated that the prognostic signature could effectively stratify patient outcomes across multiple clinical subsets, highlighting its broad applicability and independent predictive value.

Furthermore, violin plots were employed to illustrate the distribution of risk scores across these clinical stratifications (**Figures 5H–M**). With the exception of sex (**Supplementary Figure 4A**), differences in risk score distributions across cohorts stratified by age, recurrence type, WHO grade, survival status, IDH mutation, and 1p/19q codeletion were all statistically significant. These data further corroborate the robust associations between the prognostic risk scores and these critical clinical features.

### 3.6 Evaluation of the tumor immune microenvironment and mutation analysis in the high-risk and low-risk groups

To explore potential immunological correlates of the CRRG-based risk model, we characterized the tumor immune microenvironment (TIME) using computational inference methodologies. Importantly, the following analyses—including CIBERSORT, ssGSEA, and ESTIMATE—rely on the deconvolution of bulk transcriptomic profiles; thus, they reflect correlative associations rather than direct mechanistic evidence.

**Figure 6:**
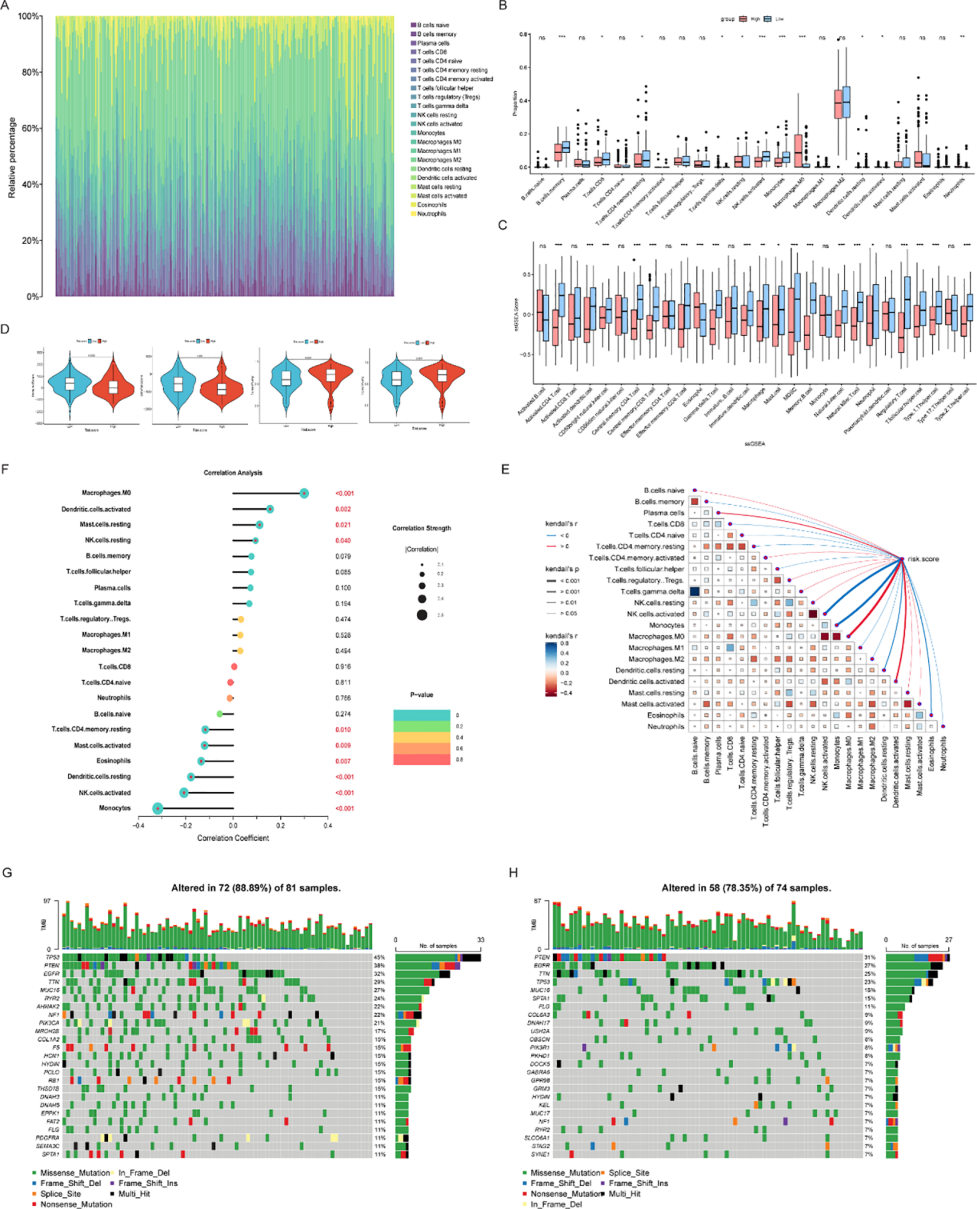
Evaluation of the tumor immune microenvironment and mutation analysis in the high- and low-risk groups. **(A)** Stacked bar chart showing the relative proportions of immune cell composition in glioma samples analyzed by the CIBERSORT algorithm; **(B)** Boxplots comparing the abundances of various immune cells between the high-risk and low-risk groups (ns, not significant; *p < 0.05, **p < 0.01, ***p < 0.001); **(C)** Boxplots depicting the differences in the immune landscape between the high-risk and low-risk groups revealed by ssGSEA (ns, not significant; *p < 0.05, **p < 0.01, ***p < 0.001); **(D)** Differences in the distributions of immune scores, stromal scores, and tumor purity between the high-risk and low-risk groups; **(E)** Correlation analysis among immune cells, as well as between immune cell infiltration and the risk score; **(F)** Correlation analysis between immune cell infiltration levels and the risk score; **(G, H)** Oncoplots (waterfall plots) displaying the mutation frequencies and types of glioma-driving genes in the high-risk and low-risk groups.

Initial assessment of the immune cell composition via CIBERSORT revealed that tumor-associated macrophages and T cells constituted the predominant immune populations within the TIME (**Figure 6A**). Notably, immune infiltration patterns differed markedly between the risk strata: the low-risk group exhibited higher abundances of activated natural killer T (NKT) cells, monocytes, and specific T and B cell subsets (**Figure 6B**).

Subsequent ssGSEA and ESTIMATE analyses further delineated distinct immune landscapes between the two groups (**Figures 6C, D**). Overall immune cell enrichment was substantially greater in the low-risk cohort, consistent with its elevated immune and stromal scores and concurrently reduced tumor purity. These observations provide a plausible immunological correlate for the more favorable clinical outcomes observed in low-risk patients.

We next evaluated the intricate correlation networks among distinct immune cell populations and their relationship with prognostic risk scores. A butterfly plot mapped these complex association patterns (**Figure 6E**): activated NKT cells exhibited a positive correlation with monocytes, and both populations correlated negatively with the risk score. This suggests a coordinated infiltration pattern that may contribute to prognostic disparities. Additionally, activated mast cells, eosinophils, and dendritic cells displayed inverse correlations with risk scores (**Figure 6F**). The concurrent depletion of these cell types as risk scores escalate, coupled with corresponding poorer patient outcomes, suggests a potential role for these populations in mediating an anti-tumor immune response.

Finally, we evaluated the tumor mutational burden (TMB) across the risk groups. *TP53* mutations were notably more frequent in the high-risk cohort (**Figure 6G**), whereas *PTEN* alterations—particularly nonsense mutations—were more prevalent in the low-risk group (**Figure 6H**), further highlighting the profound genetic heterogeneity underlying glioma progression.

### 3.7 Correlation analysis and expression validation of core genes in the prognostic model

**Figure 7:**
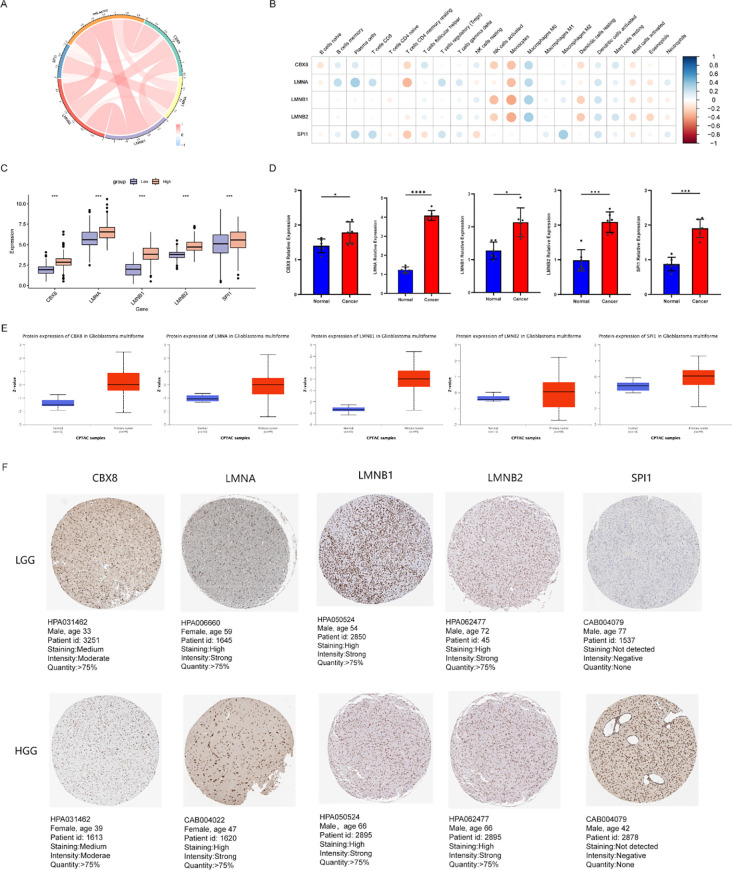
Correlation analysis and expression validation of core genes in the prognostic model. **(A)** Chord diagram illustrating the co-expression association analysis of core genes (*SPI1*, *LMNA*, *LMNB1*, *LMNB2*, *CBX8*) and the prognostic risk score; **(B)** Heatmap of the correlations between core gene expression and immune cell infiltration; **(C)** Differences in the mRNA expression of core genes between different risk groups in the CGGA_693 database (***p < 0.001); **(D)** Bar charts representing the qRT‒PCR validation results of core genes in glioma and adjacent nontumor tissues (ns, not significant; *p < 0.05, ***p < 0.001, ****p < 0.0001); **(E)** Boxplots showing the differences in protein expression of core genes between glioma and normal tissues in the UALCAN database (CPTAC data); **(F)** Representative immunohistochemical (IHC) staining images of core genes in low-grade glioma (LGG) and high-grade glioma (HGG) samples from the HPA database.

To further characterize the expression patterns and potential interrelationships among the core signature genes, we performed integrated multiomics analyses. Co-expression profiling revealed complex associations among the five core genes (*SPI1*, *LMNA*, *LMNB1*, *LMNB2*, and *CBX8*) as well as between these genes and the prognostic risk score (**Figure 7A**), suggesting possible synergistic interactions within their regulatory network. Additionally, a correlation heatmap (**Figure 7B**) quantified the strength of associations between these core genes and specific immune cell populations. As noted earlier, all core genes exhibited significant correlations with activated natural killer T (NKT) cells and monocytes.

To systematically evaluate the expression profiles of these core genes, we analyzed four independent transcriptomic datasets (CGGA_693, **Figure 7C**; CGGA_325, **Supplementary Figure 5A**; TCGA, **Supplementary Figure 5B**; GSE43378, **Supplementary Figure 5C**). These analyses revealed notable disparities in the mRNA expression levels of the core genes across risk strata, with multiple genes exhibiting significantly elevated expression in the high-risk cohort. To corroborate these *in silico* findings, we conducted qRT-PCR experiments on 6 paired glioma and adjacent non-tumor tissue samples. The results indicated that, relative to the adjacent non-tumor tissues, the mRNA levels of these core genes were markedly upregulated in the tumor samples (**Figure 7D**), suggesting that they might exert oncogenic functions during glioma initiation and progression.

To validate these findings at the translational level, we retrieved mass spectrometry-based proteomic data from the CPTAC cohort via the UALCAN platform. This analysis demonstrated that the protein expression levels of these core targets were substantially higher in glioma tissues compared to normal brain controls (**Figure 7E**), consistent with the trends observed at the transcriptomic level. Furthermore, immunohistochemical (IHC) staining data from the Human Protein Atlas (HPA) revealed that LMNA (**Figure 7F**), LMNB1, LMNB2, and CBX8 were robustly expressed across gliomas of varying grades. Notably, most of these proteins exhibited stronger staining intensities in high-grade glioma (HGG) relative to low-grade glioma (LGG), further reinforcing the potential link between these CRRGs and the malignant progression of glioma.

### 3.8 Single-cell analysis of core genes in the prognostic model

**Figure 8:**
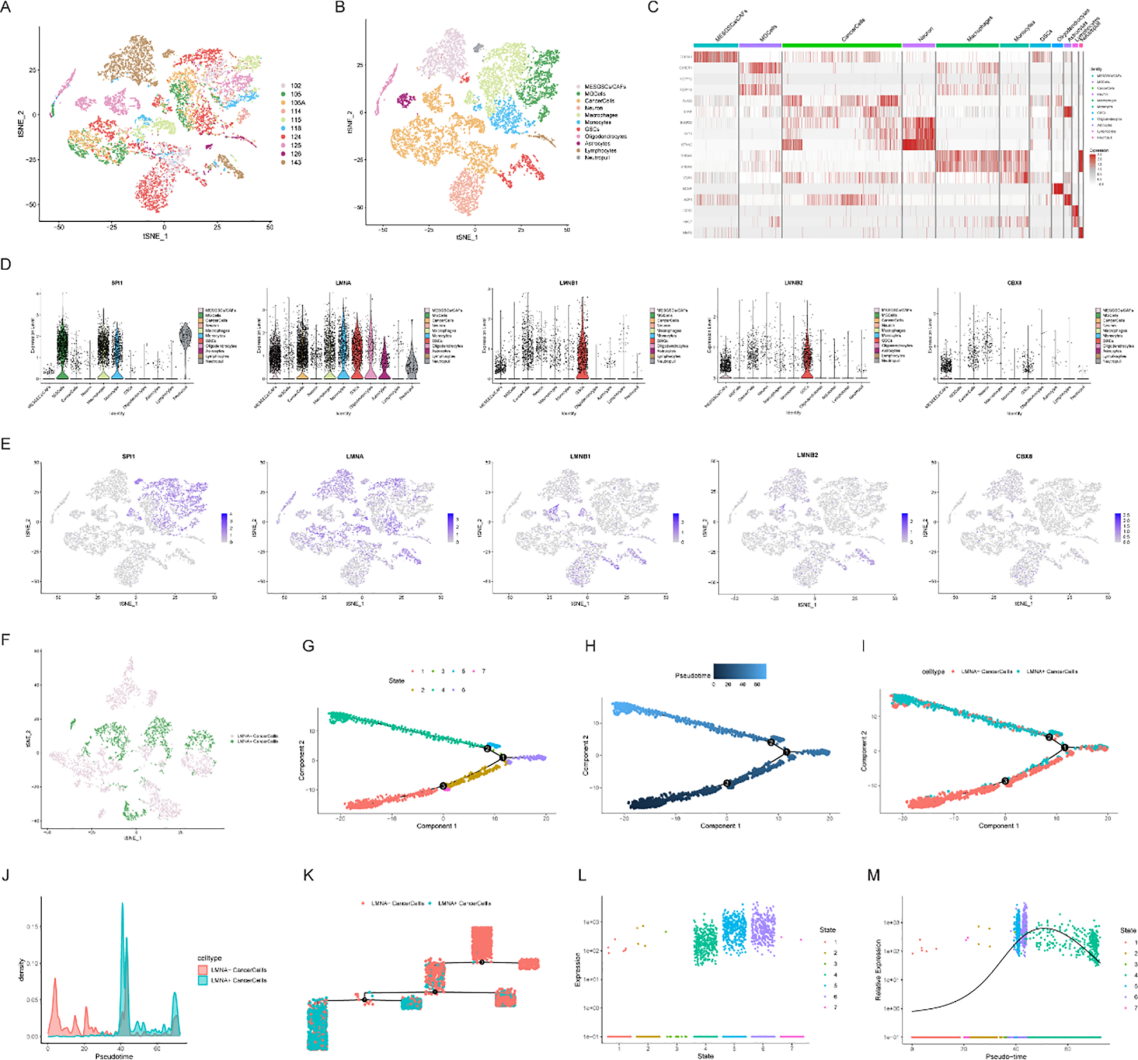
Single-cell analysis of core genes in the prognostic model. **(A)** t-SNE plot showing the distribution of cells across samples after batch correction; **(B)** t-SNE plot showing cell clustering and cell type annotation; **(C)** Heatmap of the expression profiles of cluster-specific genes; **(D)** Violin plots showing the expression differences of *SPI1*, *LMNA*, *LMNB1*, *LMNB2*, and *CBX8* across different cell types; **(E)** Spatial expression localization maps of core genes in different cell clusters; **(F)** t-SNE visualization showing the stratification of malignant cells into *LMNA*-high-expressing (*LMNA*^+^) and *LMNA*-low-expressing (*LMNA*^−^) subpopulations; **(G–K)** Inferred pseudotemporal trajectory constructed based on transcriptional similarity of malignant cell subtypes; **(L)** Scatter plot showing the expression distribution of malignant cells across different inferred cell states; **(M)** Line graph showing the expression trend of *LMNA* along the inferred pseudotime axis.

To investigate the expression patterns and regulatory characteristics of the core signature genes at the single-cell resolution, we analyzed the scRNA-seq dataset GSE131928. Standard quality control was performed utilizing the Seurat algorithm, rigorously filtering out cells with low gene counts or high mitochondrial transcript proportions (**Supplementary Figure 6A**). Subsequently, the Harmony algorithm was applied to correct for batch effects across different samples, ensuring seamless data integration (**Figure 8A**).

Following dimensionality reduction via t-SNE, the tumor microenvironment cells were segregated into 11 distinct clusters and manually annotated based on canonical cell type-specific marker genes (**Figure 8B**). The proportional distribution of these cell types varied across the individual samples (**Supplementary Figure 6B**), and a heatmap effectively delineated the expression profiles of the cluster-specific marker genes (**Figure 8C**). Violin plots further illustrated the differential expression landscapes of the five core genes (*SPI1*, *LMNA*, *LMNB1*, *LMNB2*, and *CBX8*) across the annotated cell types (**Figure 8D**). Notably, *LMNB1* and *LMNB2* were significantly enriched in glioma stem cells (GSCs). Feature plots mapping spatial expression further clarified the localization of these core genes within the tumor-associated cell clusters (**Figure 8E**).

Given that *LMNA* was significantly upregulated in bulk glioma tissues (**Figure 7D**), we sought to further elucidate its specific role within the malignant compartment. Consequently, the malignant cell subpopulation was stratified into *LMNA*-high (*LMNA*^+^) and *LMNA*-low (*LMNA*^−^) cancer cells based on their intrinsic *LMNA* expression levels (**Figure 8F**; **Supplementary Figure 6D**). A subsequent t-SNE projection revealed that these two distinct malignant subsets clustered in separate transcriptomic niches, underscoring fundamental differences in their cellular states (**Supplementary Figure 6C**).

To further contextualize this heterogeneity, we constructed a pseudotemporal trajectory to align the malignant cells along an inferred progression axis based on their transcriptional similarities (**Figures 8G–K**). Strikingly, *LMNA*^+^ cells were predominantly positioned toward the terminus of this inferred developmental trajectory, whereas *LMNA*^−^ cells clustered near its origin. Scatter plot visualization confirmed that *LMNA* expression was concentrated primarily in malignant cells assigned to States 4, 5, and 6—branches corresponding to the late/terminal segments of the pseudotime trajectory (**Figure 8L**). Consistent with these spatial mappings, a trend line demonstrated that *LMNA* expression levels climbed progressively along the inferred pseudotime axis (**Figure 8M**). This robust association suggests that elevated *LMNA* expression defines the transcriptional signature of the terminal phase of this inferred cellular progression, highlighting its potential role in driving glioma cell state dynamics.

### 3.9 Cell communication analysis and spatial transcriptomic expression of LMNA

**Figure 9:**
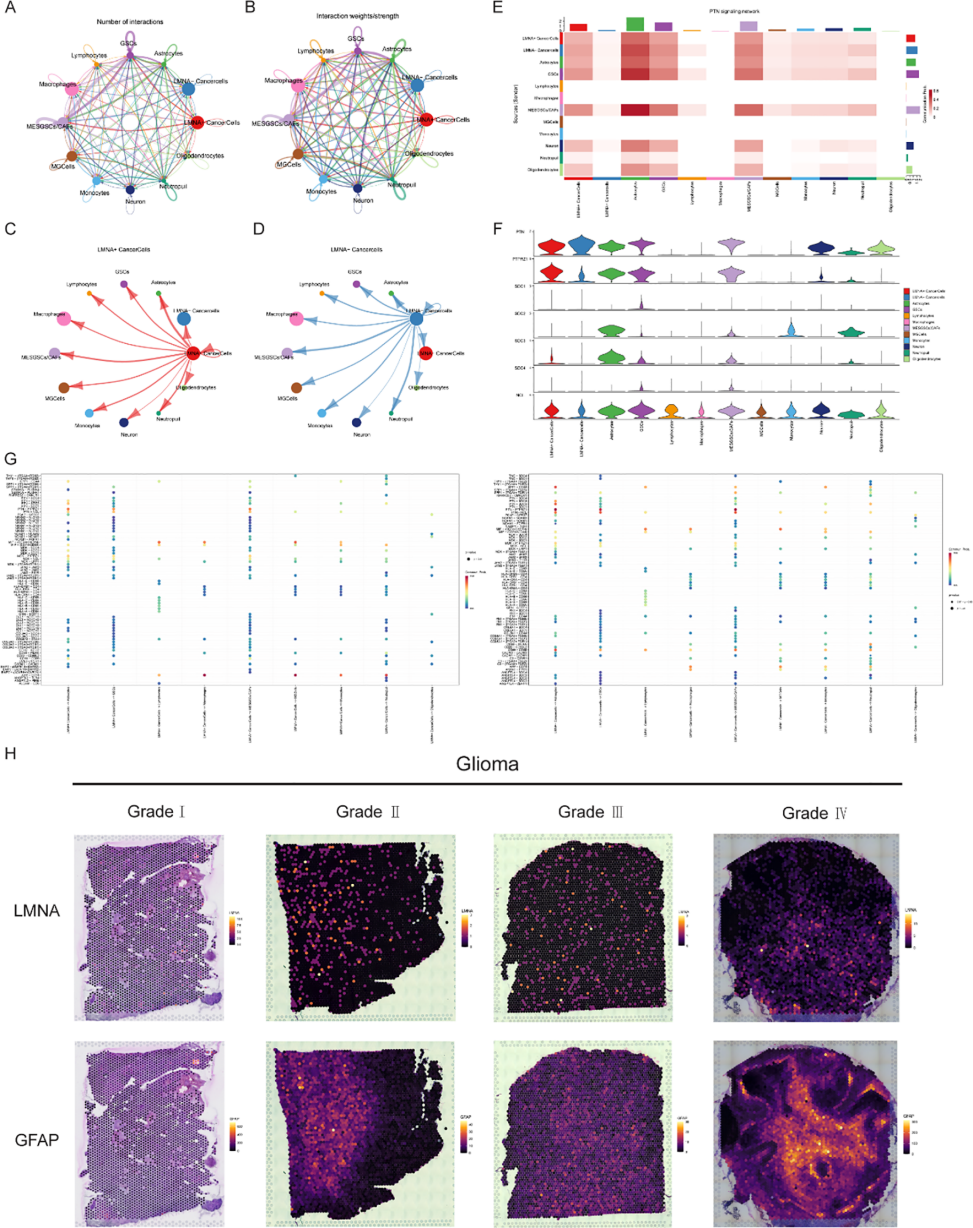
Cell Communication Analysis and Spatial Transcriptomic Expression of *LMNA*. **(A, B)** Circle plots showing the cell communication networks analyzed using the CellChat package, demonstrating significant interactions among various cell clusters; **(C, D)** Distinct interaction patterns between *LMNA*^+^ cancer cells, *LMNA*^−^ cancer cells, and other cell clusters; **(E)** Heatmap showing differences in the expression intensity of the PTN signaling pathway across cell clusters; **(F)** Violin plots displaying the differential expression of core genes in the PTN signaling pathway across different cell clusters; **(G)** Bubble plots illustrating potential ligand-receptor interactions between *LMNA*^+^ cancer cells, *LMNA*^−^ cancer cells, and other cell clusters; **(H)** Spatial expression distribution of the glioma marker glial fibrillary acidic protein (GFAP) and *LMNA* in glioma tissues of different grades (Grades I–IV).

Mapping intercellular communication networks is a fundamental component of single-cell transcriptomic research. In this study, we employed the CellChat algorithm to infer potential cell-cell communication based on the expression profiles of canonical ligand-receptor pairs (**Supplementary Figure 7A**). This analysis predicted extensive communication cross-talk among the various cell clusters within the tumor microenvironment (**Figures 9A, B**). Notably, the *LMNA*^+^ and *LMNA*^−^ malignant subsets exhibited distinctly different predicted communication patterns with neighboring cell populations (**Figures 9C, D**). Using the PTN signaling pathway as an example, the inferred signal intensity was markedly higher in the *LMNA*^+^ cancer cell cluster compared to its *LMNA*^−^ counterpart (**Figure 9E**). Correspondingly, the core genes driving the PTN pathway displayed significant differential expression between these two subpopulations (**Figure 9F**). Similarly, the inferred activities of the MK and SPP1 signaling pathways varied considerably across the cell clusters (**Supplementary Figures 7B, C**). To quantitatively compare the communication profiles of *LMNA*^+^ and *LMNA*^−^ cancer cells, scatter plots were generated to visualize their inferred interaction strengths with diverse stromal, immune, and glial cell populations (**Figure 9G**). This comparative analysis underscores fundamental differences in the predicted communication networks of the two malignant subsets, providing a theoretical basis for identifying potential *LMNA*-mediated intercellular signaling dynamics within the glioma microenvironment.

To further characterize *LMNA* expression patterns within a preserved tissue architecture, we analyzed spatial transcriptomic data from four glioma samples of varying histological grades (accession number: 35700707) utilizing the SPATA2 package. *LMNA* transcripts were highly expressed across all evaluated glioma grades and localized predominantly within the tumor core regions (**Figure 9H**), further suggesting a possible association with glioma progression and heightened malignancy.

### 3.10 LMNA promotes glioma malignancy and specific chromatin-level alterations *in vitro* and *in vivo*

**Figure 10:**
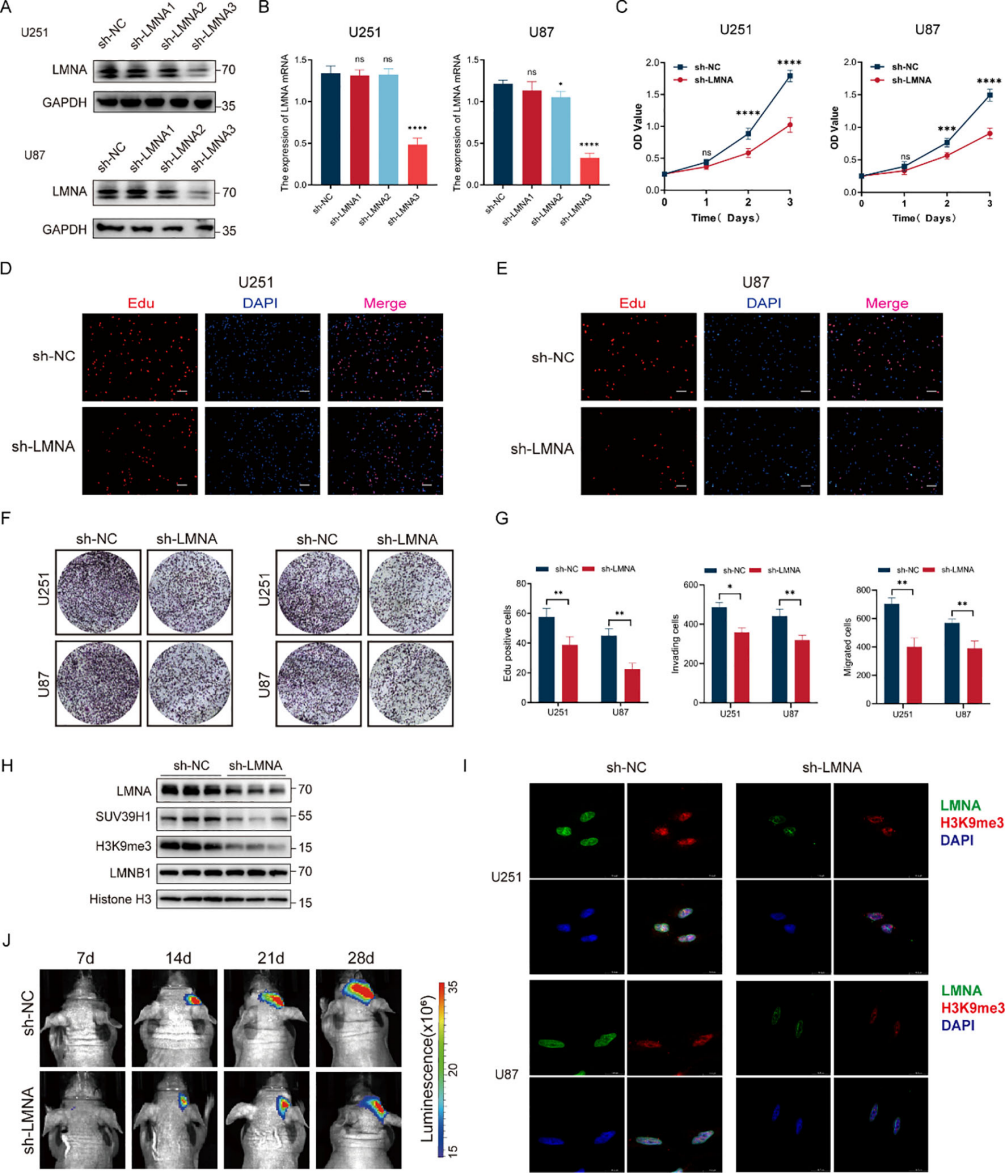
LMNA promotes glioma malignancy *in vitro* and *in vivo* and regulates the SUV39H1-H3K9me3 axis. **(A, B)** Validation of LMNA knockdown efficiency in U251 and U87 cells via Western blotting **(A)** and qRT-PCR **(B)** (n=3; ns, not significant, *p < 0.05, ****p < 0.0001). sh-LMNA3 (designated as sh-LMNA) was used for subsequent functional assays; **(C)** Cell proliferation assessed by the CCK-8 assay (n = 3 per group; ns: not significant, ***p < 0.001, ****p < 0.0001); **(D, E)** Representative images of the EdU incorporation assay in U251 **(D)** and U87 **(E)** cells (n = 3). Red: EdU; Blue: DAPI. Scale bars = 100 μm; **(F)** Representative images of Transwell assays assessing cell migration (without Matrigel) and invasion (with Matrigel) in U251 and U87 cells (n = 3). **(G)** Quantitative statistical analysis of EdU-positive cells, invading cells, and migrating cells (ns, not significant; *p < 0.05, **p < 0.01). **(H)** Western blot analysis of LMNA, SUV39H1, H3K9me3, and LMNB1 protein levels in the sh-NC and sh-LMNA groups (n = 3). Histone H3 served as the nuclear loading control. **(I)** Representative confocal immunofluorescence images of LMNA (green) and H3K9me3 (red) colocalization in U251 and U87 cells (n = 3). Blue: DAPI. Scale bars = 14.5 μm. **(J)** Representative in vivo bioluminescence images of intracranial U87 glioma xenografts at 7,14, 21, and 28 days post-transplantation (n = 5 per group).

To elucidate the biological role of LMNA in glioma progression, we established *LMNA*-knockdown cell models in two glioma cell lines (U251 and U87). Western blotting (**Figure 10A**) and qRT-PCR (**Figure 10B**) were performed to verify the knockdown efficiency. Given that sh-LMNA3 achieved the most robust suppression of LMNA expression, it was selected for all subsequent functional experiments (compared to the negative control, sh-NC).

We first evaluated the impact of LMNA depletion on the fundamental malignant behaviors of glioma cells. Compared to the sh-NC cohort, the proliferation rates of U251 and U87 cells in the sh-LMNA group were significantly reduced after 2 and 3 days of culture, as measured by the CCK-8 assay (**Figure 10C**). Furthermore, EdU incorporation assays confirmed that the proportion of highly proliferative (EdU-positive) cells markedly decreased following *LMNA* knockdown (**Figures 10D, E, G**). Transwell invasion and migration assays additionally demonstrated that the number of cells capable of penetrating Matrigel (invasion) or migrating without Matrigel was significantly lower in the sh-LMNA group than in the sh-NC group (**Figures 10F, G**), indicating that *LMNA* knockdown potently suppresses the invasion and migration capabilities of glioma cells.

Given the profound inhibitory effects of *LMNA* knockdown on tumor cell proliferation and invasion, we sought to explore the underlying molecular mechanisms, specifically focusing on its potential link to chromatin remodeling. Correlation analysis using independent datasets from the CGGA (**Supplementary Figure 8B**) and TCGA via the GEPIA platform (**Supplementary Figure 8A**) revealed that *LMNA* expression was significantly and positively correlated with core components of the Polycomb Repressive Complex 2 (PRC2), including *EZH2* and *SUZ12*, as well as with the histone methyltransferase *SUV39H1*. To experimentally test whether LMNA regulates these chromatin remodelers, we performed Western blot analysis. Following *LMNA* knockdown, the protein expression levels of EZH2, SUZ12 (**Supplementary Figure 8C**), and SUV39H1 (**Figure 10H**) were concurrently reduced in glioma cells.

To directly assess the specific chromatin-level alterations caused by LMNA loss, we evaluated the global levels of associated histone marks. Consistent with the downregulation of SUV39H1, a marked reduction in the heterochromatin-associated mark H3K9me3 was observed upon *LMNA* silencing (**Figure 10H**). Interestingly, despite the reduction in EZH2 and SUZ12 proteins, global H3K27me3 levels remained largely unchanged (**Supplementary Figure 8C**). Furthermore, LMNB1 levels showed no significant compensatory change, and Histone H3 was strictly utilized as a stable nuclear loading control (**Figure 10H**, **Supplementary Figure 8C**). These results exclude non-specific nuclear collapse and indicate that LMNA primarily and specifically modulates the SUV39H1-H3K9me3 heterochromatin axis.

To further validate the spatial connection and potential regulatory relationship between LMNA and H3K9me3, we performed confocal immunofluorescence microscopy. The results clearly demonstrated distinct nuclear colocalization of LMNA and H3K9me3. Importantly, consistent with our Western blot findings, the fluorescence intensity of H3K9me3 was substantially diminished following *LMNA* silencing (**Figure 10I**), providing direct visual evidence of LMNA’s crucial role in maintaining H3K9me3-associated heterochromatin integrity.

Finally, to validate the oncogenic role of LMNA *in vivo*, we performed intracranial orthotopic transplantation of U251 cells expressing sh-NC or sh-LMNA in nude mice. *In vivo* bioluminescence imaging (**Figure 10J**) revealed that the tumor fluorescence signal intensity in the sh-LMNA group was significantly weaker than that in the sh-NC group at days 7, 14, 21, and 28. Quantitative analysis (**Supplementary Figure 8D**) further confirmed that the bioluminescence signal of tumors in the sh-LMNA group was notably lower than that in the sh-NC group, firmly suggesting that *LMNA* knockdown effectively inhibits glioma growth *in vivo*.

### 3.11 Drug screening and molecular docking targeting LMNA

**Figure 11:**
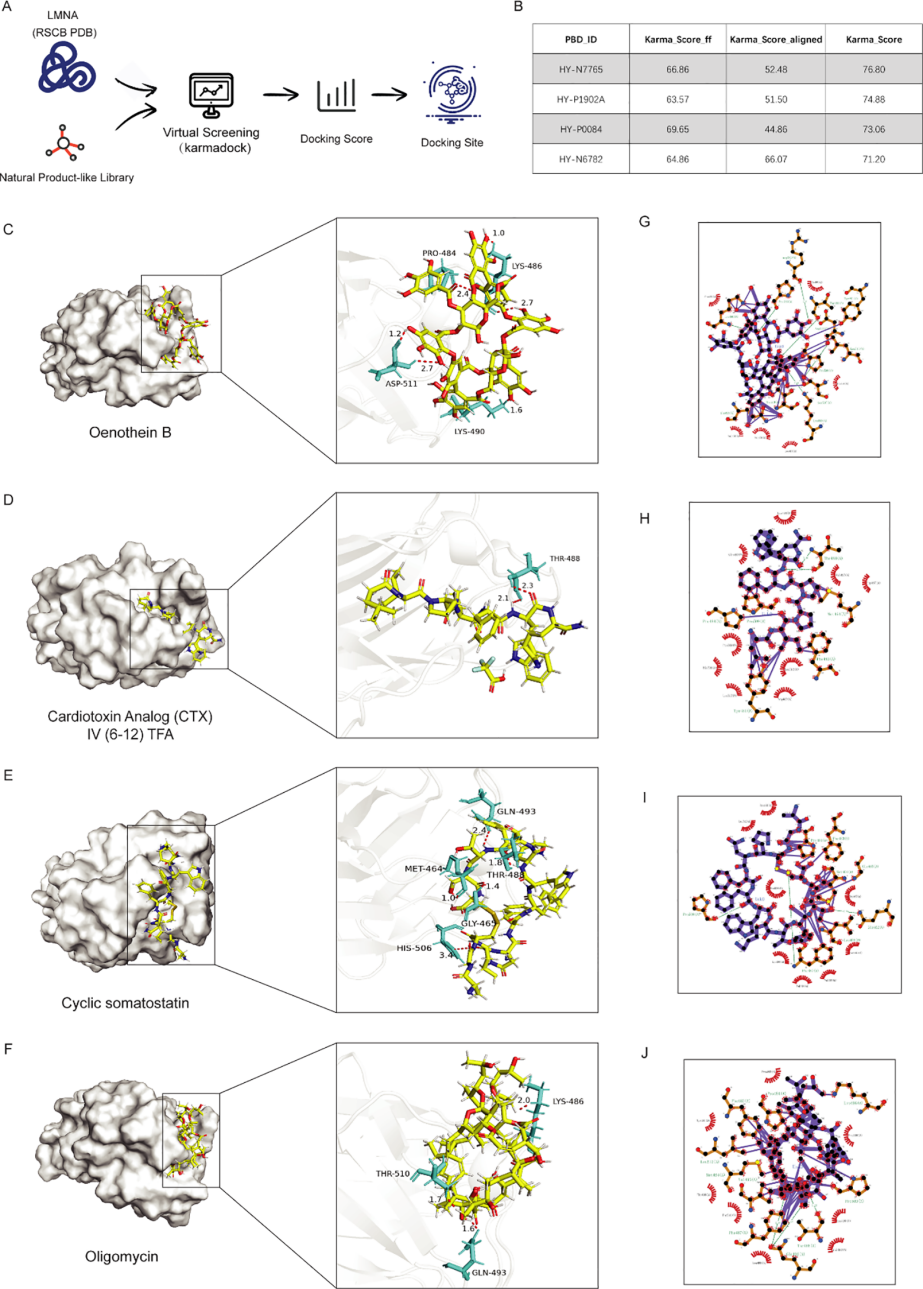
Virtual screening and molecular docking verification of potential therapeutic drugs that target LMNA. **(A)** Schematic diagram of the virtual screening process using KarmaDock, which is based on the LMNA domain from the RCSB PDB database and the MCE Natural Product Library; **(B)** Table summarizing the information of the 4 high-scoring natural product small molecules (all scores > 70) selected by the Karma_Score ranking, including the PDB identification code (PDB_ID) and Karma_Score; **(C–F)** Three-dimensional molecular docking results of Oenothein B **(C)**, cardiotoxin analog (CTX) IV (6-12), TFA **(D)**, cyclic somatostatin **(E)**, and oligomycin **(F)** with LMNA; **(G–J)** Two-dimensional interaction diagrams of the small molecules docked with LMNA, displayed via the LigPlus tool.

To explore potential therapeutic strategies targeting LMNA, we performed *in silico* virtual screening to identify candidate compounds. The three-dimensional structure of the LMNA domain was retrieved from the RCSB Protein Data Bank (PDB), and the MCE Natural Product Library was screened utilizing the KarmaDock pipeline (**Figure 11A**). Based on the Karma_Score rankings, the top four natural product molecules (all scoring > 70) were identified (**Figure 11B**): Oenothein B (HY-N7765), Cardiotoxin Analog (CTX) IV (6–12) TFA (HY-P1902A), Cyclic somatostatin (HY-P0084), and Oligomycin (HY-N6782). Among these, Oenothein B—a poly(ADP-ribose) glycohydrolase inhibitor with diverse reported pharmacological activities, including anti-tumor effects—achieved the highest docking score. Subsequent three-dimensional visualization using PyMOL (**Figures 11C–F**) and two-dimensional interaction analysis using LigPlus (**Figures 11G–J**) revealed favorable predicted binding modes for all four compounds with the LMNA target. Importantly, these computational findings provide a preliminary basis for hypothesizing potential LMNA-targeting compounds and serve to nominate candidates for future experimental validation, rather than establishing a definitive therapeutic priority.

## 4 Discussion

Glioma remains a major oncological challenge characterized by a poor prognosis, underscoring the urgent need for improved therapeutic strategies and prognostic tools (1). This study integrates multiomics data with 117 machine learning algorithms to systematically explore the role of chromatin remodeling in glioma subtyping, prognostic evaluation, and molecular mechanisms.

This work constitutes the first comprehensive assessment of the role of chromatin remodeling in glioma. Initially, through differential expression analysis across a large cohort of samples, we identified genes significantly dysregulated in glioma. The functions and pathways enriched among these differentially expressed genes were closely associated with glioma cell proliferation and immune response regulation, suggesting their potential involvement in glioma initiation and progression. Subsequently, by leveraging chromatin remodeling-related genes (CRRGs), we conducted consensus clustering on glioma patients. The *k* = 2 threshold emerged as the optimal configuration, stratifying patients into two transcriptionally and clinically distinct subgroups (Subgroup 1 and Subgroup 2). Analyses utilizing the CIBERSORT, ssGSEA, and ESTIMATE algorithms revealed pronounced differences in immune infiltration between these strata. Subgroup 2 exhibited greater immune cell infiltration and a superior prognosis. Enrichment analysis indicated that these two subgroups and their corresponding DEGs were strongly linked to DNA/RNA binding and chromatin remodeling activities.

We next identified 28 glioma-specific DECRRGs and constructed prognostic models using 117 machine learning approaches. The SuperPC-based model demonstrated optimal predictive performance, effectively stratifying patients into high- and low-risk groups with significant survival disparities, which were validated across multiple independent cohorts (TCGA, GEO, CGGA). ROC analysis yielded AUCs of 0.75, 0.66, and 0.77 for 1-, 3-, and 5-year survival, respectively, confirming its predictive accuracy. Importantly, this model was selected based on consistent performance across two independent validation cohorts, thereby minimizing overfitting and ensuring broad generalizability.

Immune infiltration analysis delineated distinct tumor immune microenvironment (TIME) landscapes between the risk groups. The low-risk cohort exhibited higher levels of B cells, CD8+ T cells, activated NKT cells, and M0 macrophages, implicating the immune microenvironment in glioma progression and suggesting potential immunotherapeutic vulnerabilities.

The prognostic signature comprises five core genes (*SPI1*, *LMNA*, *LMNB1*, *LMNB2*, *CBX8*), all of which have been previously linked to glioma progression. *SPI1* activates the PI3K/AKT pathway to promote GSC transition (35, 36). LMNA, LMNB1, and LMNB2 contribute to cell proliferation, migration, and cell cycle regulation via PI3K/AKT signaling and pRb-mediated arrest (37, 38). *CBX8* enhances proliferation and invasion while reducing radiosensitivity through DNA damage repair pathways (39). Collectively, these genes are robustly associated with patient survival and tumor immunity.

Multiomics validation (qRT-PCR, proteomics, spatial transcriptomics) corroborated the upregulation of these core genes in glioma. Single-cell analysis further unveiled distinct spatial distributions: *SPI1* was enriched in myeloid cells, *LMNB1*/*LMNB2* in GSCs, and *LMNA* was broadly expressed. Pseudotime and cell communication analyses implicated *LMNA* in glioma progression and intercellular TME crosstalk.

LMNA, which encodes lamin A/C, structurally maintains the nuclear envelope, chromatin organization, and genomic stability (40). It regulates DNA damage repair (41), contributing to chemoresistance (42, 43), and modulates TME remodeling, mitochondrial function, and metabolic reprogramming via diverse signaling pathways (44, 45). In glioma, elevated *LMNA* expression correlates with a poor prognosis (38) and promotes proliferation and invasion via the PI3K/AKT axis (37). Our data confirmed *LMNA* as a core gene within the prognostic model, with elevated expression consistently observed in high-risk patients. Single-cell transcriptomics showed *LMNA* enrichment in malignant cells at late differentiation stages and co-expression with *SPI1*/*CBX8*, suggesting a coordinated role in chromatin regulation.

Functional experiments rigorously validated the oncogenic role of LMNA in glioma. *LMNA* knockdown significantly impaired tumor cell proliferation, migration, and invasion *in vitro*, and markedly suppressed intracranial tumor growth *in vivo*. Beyond these profound phenotypic effects, our study uncovers a highly specific mechanistic link between LMNA and epigenetic reprogramming. While LMNA structurally maintains nuclear architecture, its enrichment in late-stage malignant cells and its strong correlation with epigenetic modifiers suggest a more active regulatory role in chromatin remodeling.

Specifically, our bioinformatic analyses revealed positive correlations between *LMNA* and PRC2 components (*EZH2*, *SUZ12*) as well as the heterochromatin-associated histone methyltransferase *SUV39H1*. Strikingly, our functional validation unmasked a preferential vulnerability of the SUV39H1-H3K9me3 axis to LMNA loss. Following *LMNA* knockdown, the expression of SUV39H1 and the global levels of its associated heterochromatin mark, H3K9me3, were drastically reduced. This was further reinforced by immunofluorescence assays, which vividly demonstrated the precise spatial colocalization of LMNA with H3K9me3 in the nucleus and confirmed the concurrent loss of the H3K9me3 signal upon LMNA depletion. This direct protein and spatial evidence strongly indicates that LMNA is indispensable for maintaining H3K9me3-associated heterochromatin integrity in glioma.

Interestingly, while *LMNA* silencing successfully downregulated the PRC2 core components EZH2 and SUZ12, the global levels of H3K27me3 remained largely unaltered. This uncoupling may reflect the high stability of the H3K27me3 mark, potential compensation by homologous methyltransferases (e.g., EZH1), or locus-specific redistributions that require future high-resolution ChIP-seq profiling. However, this precise selectivity—specifically impairing the SUV39H1-H3K9me3 axis while sparing global H3K27me3—powerfully argues against non-specific nuclear envelope collapse. Furthermore, the absence of compensatory alterations in LMNB1, alongside consistent nuclear loading confirmed by Histone H3, further dispels concerns of generalized structural failure. Collectively, this multi-level evidence refines the narrative of LMNA from a mere structural scaffold to a crucial epigenetic driver that sustains glioma malignancy through targeted heterochromatin maintenance.

This study has certain limitations. First, cross-cohort comparisons (e.g., GTEx vs. CGGA) may introduce inherent batch effects. Second, while validated across multiple datasets, the model may not fully capture global glioma heterogeneity. Third, the direct mechanistic link and binding interfaces between LMNA and chromatin remodelers warrant deeper molecular investigation. Fourth, the candidate compounds identified via virtual screening require comprehensive experimental validation.

In summary, we established a robust chromatin remodeling-related prognostic model and identified LMNA as a potential biomarker and therapeutic target in glioma. Preliminary mechanistic data linking LMNA to Polycomb/SUV39H1-mediated epigenetic regulation provide a solid theoretical foundation for future investigations.

## 5 Conclusions

In summary, this study systematically analyzed the expression characteristics of chromatin remodeling-related genes (CRRGs) in glioma and, based on specific DECRRGs, stratified patients into two distinct subtypes (Subgroup 1 and Subgroup 2) with significantly different prognostic trajectories. Notably, patients in Subgroup 1 experienced poorer clinical outcomes, characterized molecularly by dysregulated chromatin remodeling activity, blunted immune cell infiltration, and a deeply imbalanced tumor microenvironment. By systematically evaluating 117 machine learning frameworks, a SuperPC-based ensemble signature was ultimately identified as the optimal prognostic model following a stringent multi-cohort validation pipeline. The CRRG-derived prognostic model constructed herein not only deepens our understanding of how chromatin remodeling dictates glioma progression but also offers a robust clinical tool for personalized prognostic assessment and therapeutic decision-making.

Furthermore, our findings highlight LMNA as a potential novel oncogenic driver and a promising prognostic biomarker in glioma. Its multifaceted role extends far beyond merely promoting cellular proliferation, invasion, and *in vivo* tumor growth. Crucially, our newly integrated data link elevated *LMNA* expression to the Polycomb chromatin regulatory network and the SUV39H1-H3K9me3 axis, suggesting a potential mechanism by which LMNA may actively reshape the epigenetic landscape and transcriptional programs to fuel glioma malignancy. This profound epigenetic involvement underscores the significance of LMNA not only as a critical prognostic indicator but also as a highly promising node for targeted therapeutic intervention.

## Data Availability

Publicly available datasets were analyzed in this study. This data can be found here: https://www.gsea-msigdb.org/gsea/index.jsp, https://ualcan.path.uab.edu/, https://www.proteinatlas.org/.
