# Supplementary material for: Integrating multiomics to elucidate the role of chromatin remodeling in glioma and the antitumor mechanisms and therapeutic potential of targeting LMNA
Source: Front Immunol. 2026 May 8;17:1759811. doi: 10.3389/fimmu.2026.1759811 (PMC13194460; doi:10.3389/fimmu.2026.1759811)
Supplement: Supplementary file 1 [file DataSheet1.docx]

**
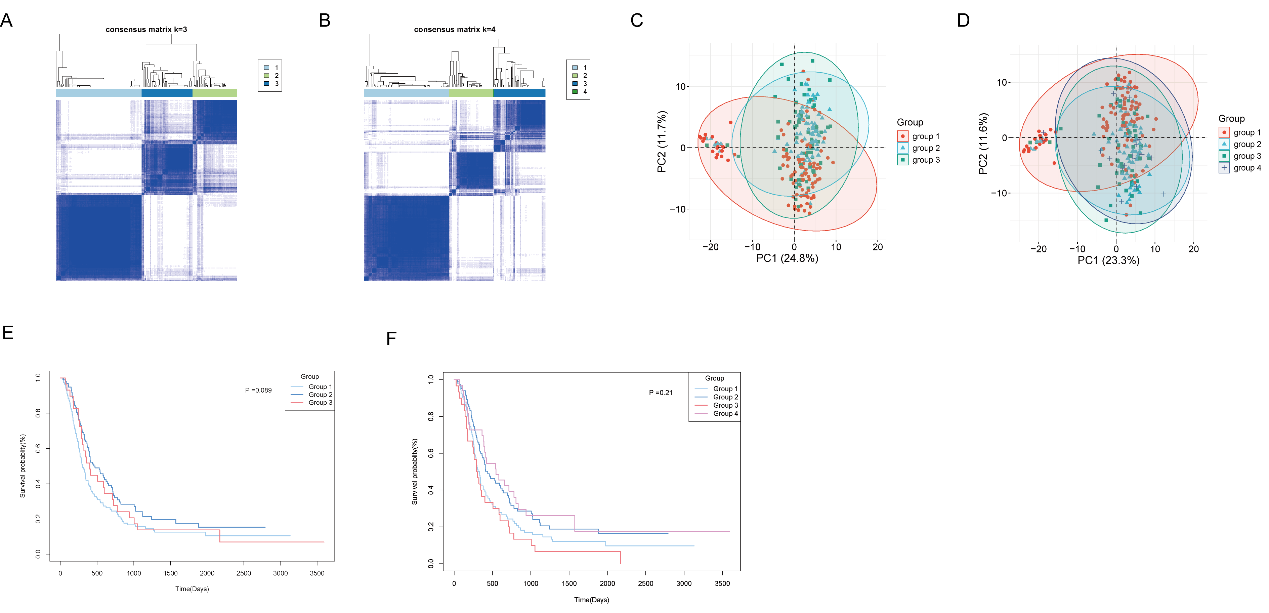
**

**Supplementary Figure 1**

A, B: Consensus matrices for k=3 (A) and k=4 (B) show poor stability and indistinct boundaries.

C, D: When K=3/4, principal component analysis (PCA) demonstrates the degree of separation among subgroups.

E, F: When K=3/4, survival analysis was performed to compare the outcomes among the subgroups.

**
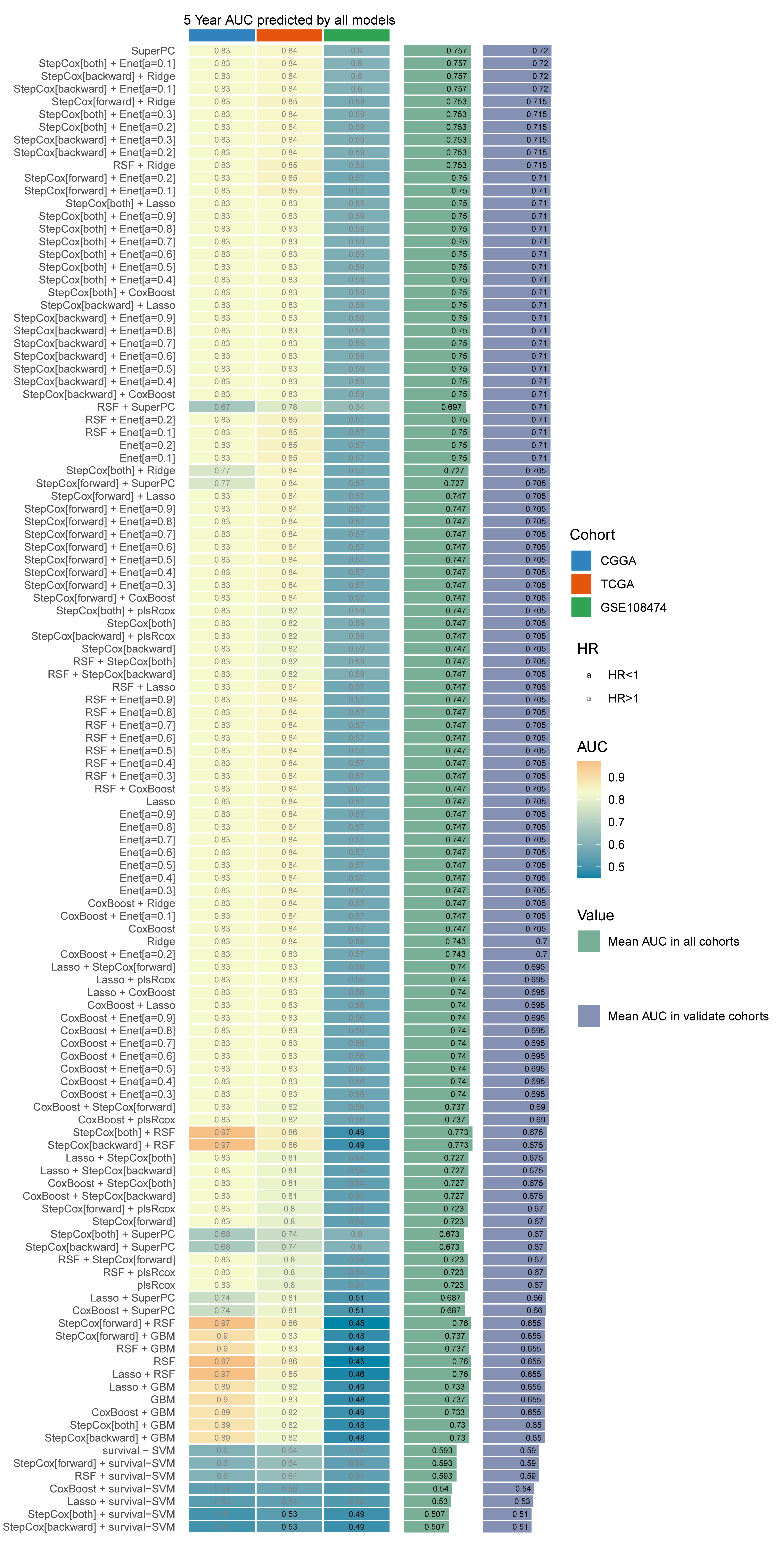
**

**Supplementary Figure 2**

To objectively compare and select the optimal prognostic model, we calculated the AUC (5‑year overall survival) of each model across the 117 combined algorithms generated by 10 machine learning approaches.


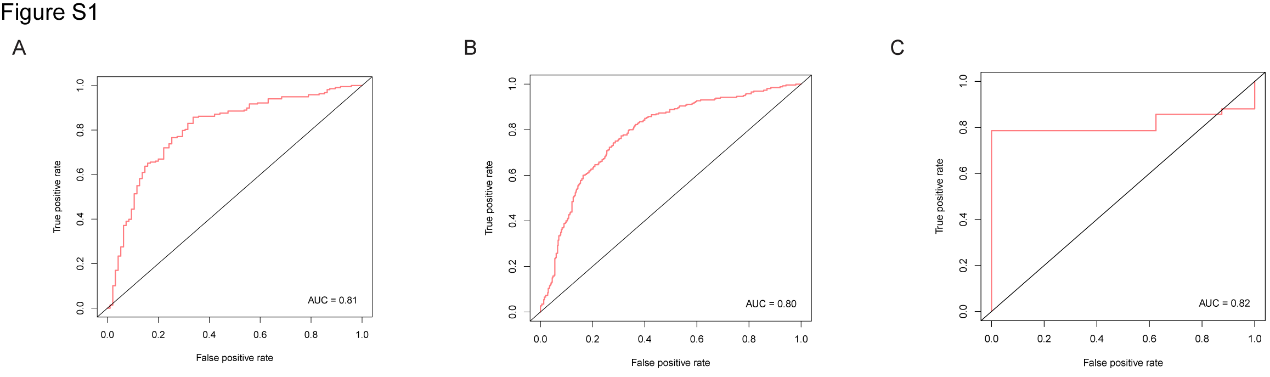


**Supplementary Figure 3**

A–C: Time-dependent ROC curves for overall survival in the CGGA_325, TCGA, and GSE43378 datasets.


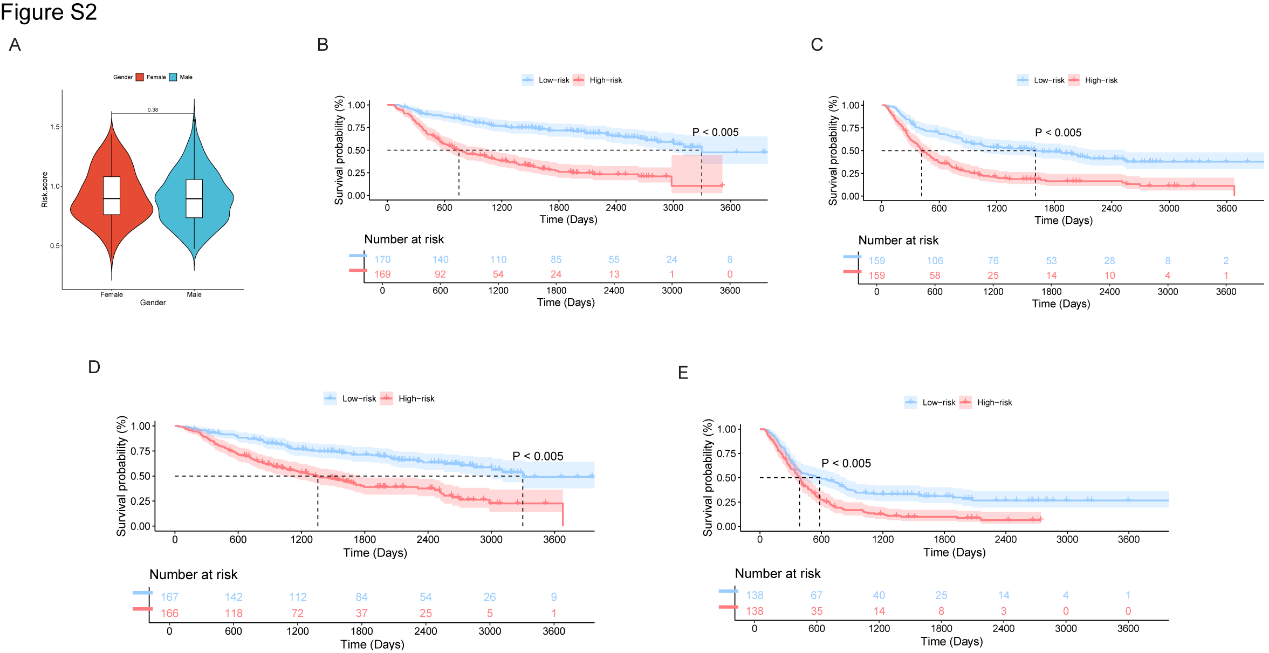


**Supplementary Figure 4**

A: Violin plot showing the distribution of risk scores across different sex subgroups;

B, C: Survival curves of high-risk and low-risk patients in different age subgroups;

D, E: Survival curves of high-risk and low-risk patients in different IDH mutation status subgroups.


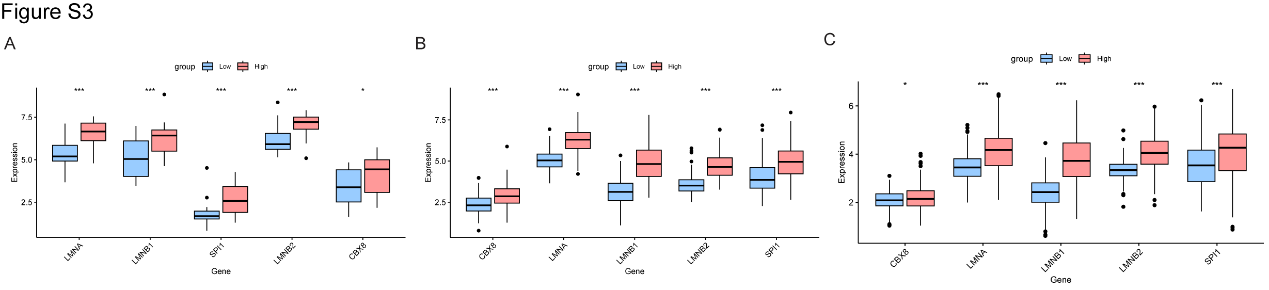


**Supplementary Figure 5**

A: Boxplots showing the differential mRNA expression of core genes between different risk groups in the CGGA_325 database;

B: Boxplots showing the differential mRNA expression of core genes between different risk groups in the TCGA database;

C: Boxplots showing the differential mRNA expression of core genes between different risk groups in the GSE43378 database.


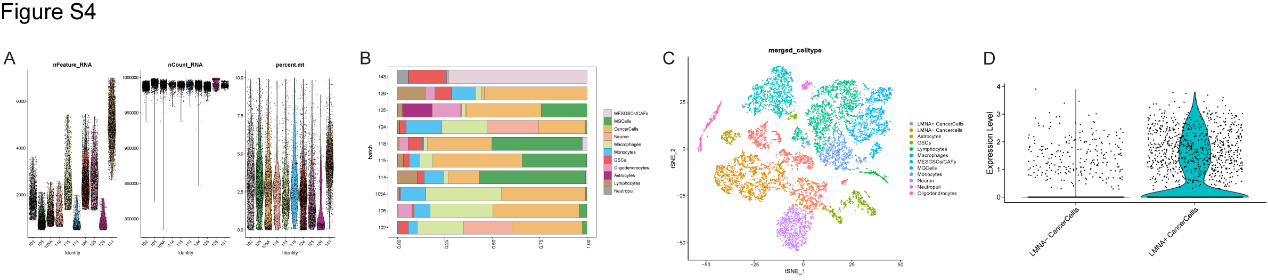


**Supplementary Figure 6**

A: Quality control metrics of single-cell RNA-seq data across samples;

B: Differences in the distribution of cell types across different samples;

C: t-SNE plot showing the distribution of LMNA-high/low-expressing malignant cells;

D: Differences in LMNA expression levels in malignant cell subpopulations.


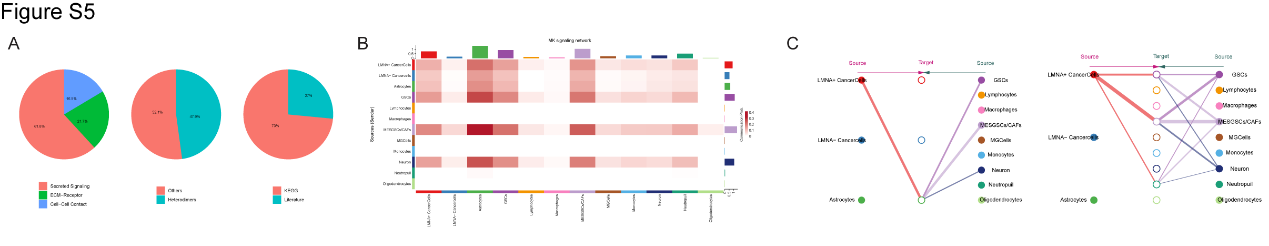


**Supplementary Figure 7**

A: Pie charts showing the composition of cell communication analyzed by the CellChat package;

B: Heatmap showing the differential expression of the MK signaling pathway across different cell clusters;

C: Hierarchy network plots showing the differential expression of the SPP1 signaling pathway across different cell clusters.


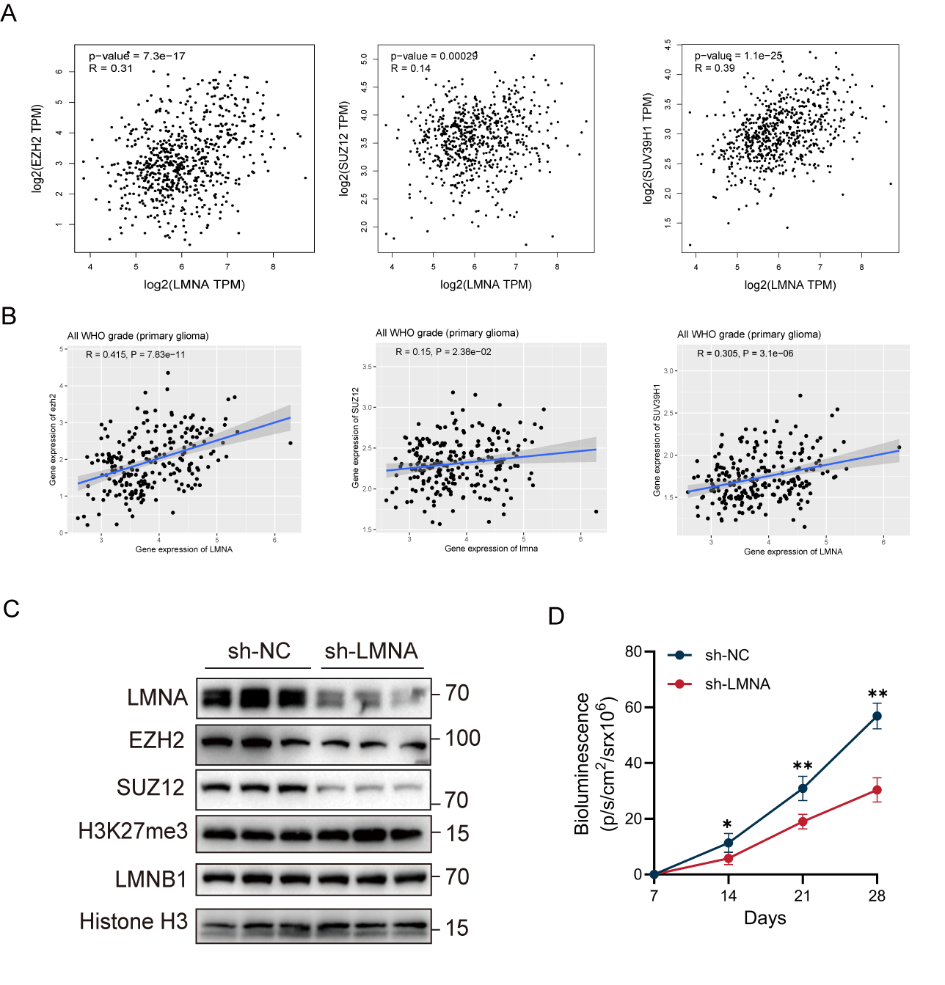


**Supplementary Figure 8**

A, B: Scatter plots showing correlation analysis between LMNA expression and the core epigenetic regulators EZH2, SUZ12, and SUV39H1 in glioma, utilizing the TCGA dataset via the GEPIA platform (A) and the CGGA dataset (B).

C: Western blot analysis of LMNA, EZH2, SUZ12, H3K27me3, and LMNB1 protein levels in the sh-NC and sh-LMNA groups. Histone H3 served as the stable nuclear loading control.

D: Line graph showing the quantitative statistical analysis of the in vivo bioluminescence intensity of intracranial glioma xenografts(n=5) over a 28-day period (*P < 0.05, **P < 0.01).
